# Chronic liver disease is an important risk factor for worse outcomes in acute pancreatitis: a systematic review and meta-analysis

**DOI:** 10.1038/s41598-024-66710-w

**Published:** 2024-07-19

**Authors:** Jakub Hoferica, Ruben Zsolt Borbély, Ali Nedjati Aghdam, Eszter Ágnes Szalai, Ádám Zolcsák, Dániel Sándor Veres, Krisztina Hagymási, Bálint Erőss, Péter Hegyi, Peter Bánovčin, Péter Jenő Hegyi

**Affiliations:** 1 Centre for Translational Medicine, Semmelweis University, Budapest, Hungary; 2 Clinic of Internal Medicine - Gastroenterology, Jessenius Faculty of Medicine in Martin, Comenius University, Bratislava, Slovakia; 3 Department of Medical Imaging, Bajcsy-Zsilinszky Hospital and Clinic, Budapest, Hungary; 4 Department of Restorative Dentistry and Endodontics, Semmelweis University, Budapest, Hungary; 5 Department of Biophysics and Radiation Biology, Semmelweis University, Budapest, Hungary; 6 Department of Surgery, Transplantation and Gastroenterology, Semmelweis University, Budapest, Hungary; 7 Institute of Pancreatic Diseases, Semmelweis University, Budapest, Hungary; 8 Institute for Translational Medicine, Medical School, University of Pécs, Pécs, Hungary; 9 Translational Pancreatology Research Group, Interdisciplinary Centre of Excellence for Research Development and Innovation, University of Szeged, Szeged, Hungary

**Keywords:** Acute pancreatitis, Cirrhosis, Steatotic liver disease, Nonalcoholic fatty liver disease, Metabolic-associated fatty liver disease, Acute pancreatitis, Liver diseases

## Abstract

Chronic liver diseases (CLD) affect 1.5 billion patients worldwide, with dramatically increasing incidence in recent decades. It has been hypothesized that the chronic hyperinflammation associated with CLD may increase the risk of a more severe course of acute pancreatitis (AP). This study aims to investigate the underlying impact of CLD on the outcomes of AP. A systematic search was conducted in Embase, Medline, and Central databases until October 2022. Studies investigating patients with acute pancreatitis and CLD, were included in the meta-analysis. A total of 14,963 articles were screened, of which 36 were eligible to be included. CLD was a risk factor for increased mortality with an odds ratio (OR) of 2.53 (CI 1.30 to 4.93, *p* = 0.01). Furthermore, renal, cardiac, and respiratory failures were more common in the CLD group, with ORs of 1.92 (CI 1.3 to 2.83, *p* = 0.01), 2.11 (CI 0.93 to 4.77, *p* = 0.062) and 1.99 (CI 1.08 to 3.65, *p* = 0.033), respectively. Moreover, the likelihood of developing Systemic Inflammatory Response Syndrome (SIRS) was significantly higher, with an OR of 1.95 (CI 1.03 to 3.68, *p* = 0.042). CLD is an important risk factor for worse outcomes in AP pancreatitis, leading to higher mortality and increased rates of local and systemic complications.

## Introduction

Acute pancreatitis (AP), characterized by a variable and hard-to-predict course, ranks as the third most common cause of hospitalization in gastroenterology^1^. Mild forms of AP exhibit a mortality rate of less than 1% and short hospitalization time, whereas severe cases can reach mortality rates from 20 to 40% and much longer average hospitalization time^2–4^. To ensure the effective management of patients with AP, it is crucial to identify the risk of adverse outcomes throughout the disease course early on. However, the current prognostic models have limited clinical applicability, underscoring the need for novel prognostic indicators^5,6^.

Chronic liver disease (CLD) is a prevalent global health issue that has shown an increasing trend over the past two decades, affecting approximately 1.5 billion individuals^7^. This increase in prevalence can be attributed to the widespread epidemic of obesity, high alcohol consumption, and the high prevalence of hepatotropic viruses. Notably, causes and risk factors for CLD and AP overlap significantly, making these diseases prone to coexistence, especially in the case of alcohol-related diseases and alcohol-induced AP. CLD encompasses a diverse group of diseases characterized by gradual fibrosis development and progression toward advanced and end-stage CLD. Moreover, CLD is associated with a higher risk of cardiovascular diseases, chronic kidney diseases, obesity, malnutrition, sarcopenia, and frailty, predisposing a patient to worse outcomes^8–10^. Although CLD, at all its stages, has been associated with worse outcomes in various diseases, the available data on the relationship between AP and CLD remain insufficient. Usually, patients with AP undergo at least one imaging method, such as abdominal ultrasound, magnetic resonance, or computed tomography, which can also detect the presence of many forms of CLD. Consequently, CLD could also serve as a valuable prognostic factor for evaluating the severity of AP.

Even though some authors report the adverse effects of specific liver diseases^11^ on the outcomes of AP, a comprehensive systematic review or meta-analysis investigating the association between AP and CLD is currently lacking. Therefore, this study aims to fill this knowledge gap and better understand the relationship between the two conditions.

## Results

### Search and selection

**Figure 1:**
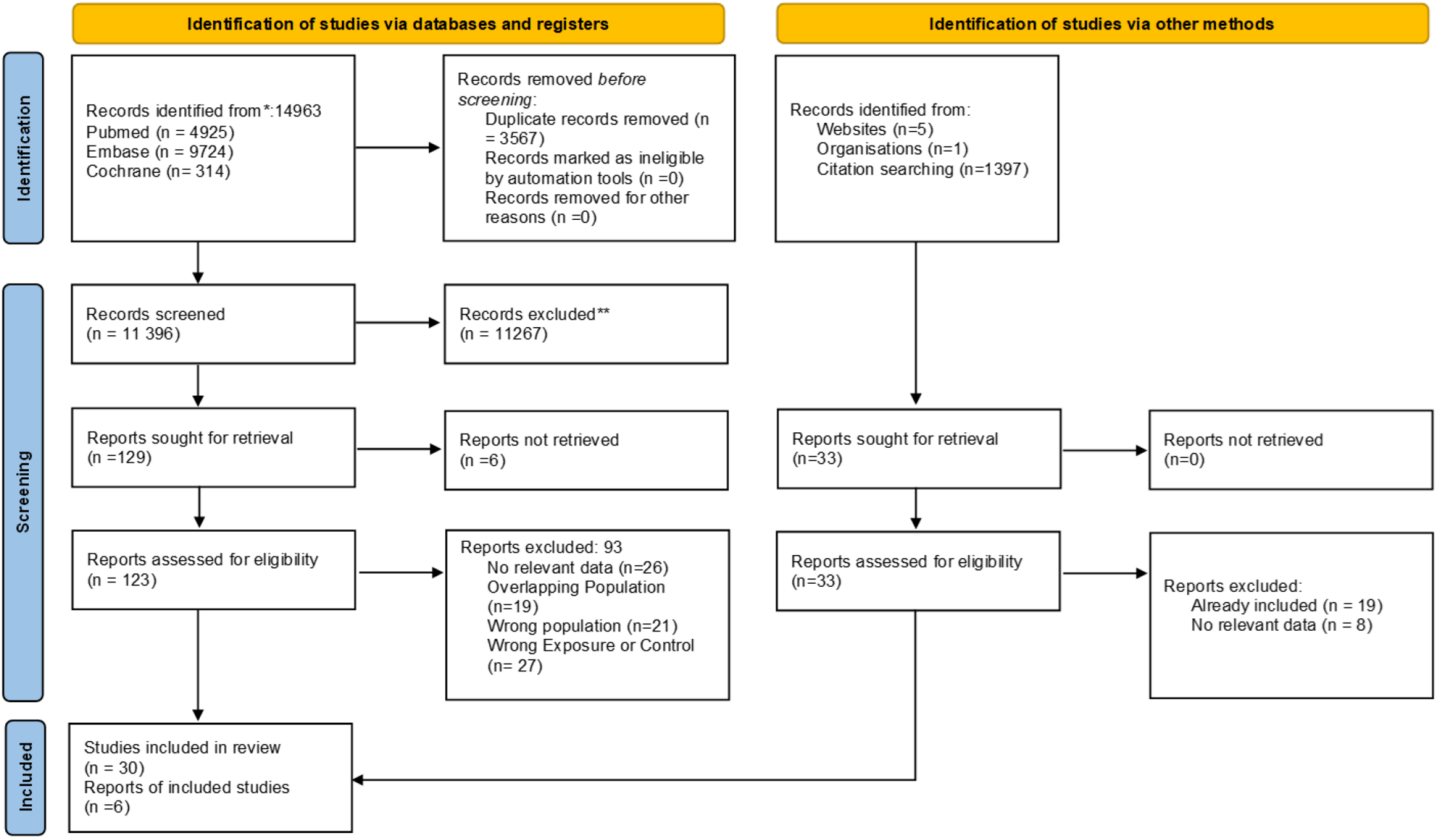
Prisma 2020 flow diagram of the screening and selection process.

A total of 14,963 publications were identified for the selection process. After duplicate removal, title, and abstract selection, 123 remaining publications underwent full-text selection. Cohen’s Kappa was 0.80 for title and abstract and 0.92 for full-text selection. Thirty^11–40^ of the 123 publications were eligible to be included in the review. Six additional publications^41–46^ were included using citation search^47^ and website search. One study data were acquired from our institution in the preprint state^44^; for details, see the Prisma flowchart (Fig. 1).

The issue of overlapping populations was raised when data from database-based studies were pooled^23,30,33,34^. It was determined that only one database study should be included in the one synthesis. If more than one study reported on the same outcome, all of them were excluded from the synthesis to avoid any potential selection bias.

### Basic characteristics of studies included

Of the 36 included publications, 12 reported cirrhosis^12,14,18,20,22,23,26,31–35^, and 24 reported steatotic liver disease (SLD)^11,13,16–19,21,24,25,27–30,36–46^, seven of which reported Nonalcoholic fatty liver disease (NAFD)^19,25,30,37,39,41,45^ and one Metabolic-associated fatty liver disease (MAFDL)^44^, the remaining publications did not specify the type of fatty liver. Five publications employed the International Classification of Diseases codes (ICD) to diagnose AP, whereas the rest used two of the three criteria according to the revised Atlanta criteria^48^. See Table 1 for further details of the baseline characteristics of each study.

**Table 1:** Main characteristics of the included studies in the systematic review and meta-analysis.

Authors	Year	Design	Country (study period)	Population	Age (years) Mean (SD)^a^	Female (%)	AP definition	CLD type	CLD diagnosis	Outcomes
Xu et al.	2015	Retrospective	China (2000–2014)	2671	50.965 (17.35)	40.5	2/3	ALFD/NAFLD	CT Liver/spleen ratio ± alcohol intake	Severity, Systemic Local complications, ANC, Infection, SIRS, Renal, Cardiac Respiratory failure CRP, APACHE-II
Liu et al.	2022	Retrospective	USA 2001–2021	631	60.38 (47.08–72.54)	44.9	2/3	Cirrhosis	N/A	Mortality
Liu et al.	2022	Retrospective	China (2016- 2021)	186	53.06 (15.33)	35	2/3	Fatty liver	CT, US, MRI	Severity, ANC, SIRS, APACHE, LOS, CRP
Brivet et al.	1999	Prospective	France (1991–1993)	50	53.8 (12)	33	2/3	Cirrhosis	N/A	Mortality
Ding et al.^b^	2018	Retrospective	China (2014–2016)	142	55.87 (15.47)	47.8	2/3	Fatty liver	N/A	IPN
Frey et al.	2007	Retrospective	USA (1992–2001)	84,713	54.5 (N/A)	53.9	ICD	CLD	ICD	MODS
Gupta et al.	2021	Retrospective	India (2017–2019)	103	39.53 (12.73)	22.1	2/3	Fatty liver	CT	Mortality, Organ failure, MODS
Jang et al.	2021	Retrospective	South Korea (2011–2020)	242	47 (12.6)	11.2	2/3 presumed AAP	Fatty liver Cirrhosis	CT Liver/spleen laboratory clinical finding	Severity, Renal, Cardiac, and Respiratory failure
Mikolasevic et al.	2016	Retrospective	Croatia (2018–2015)	822	63.9 (17)	51.1	2/3	NAFLD	CT	Mortality, Severity, AFC, ANC, PC, Organ failure, MODS, LOS, CRP, APACHE-II
Park et al.	2019	Retrospective	South Korea (2008–2017)	672	N/A	N/A	2/3	Cirrhosis	N/A	Severity
Roussey et al.^b^	2022	Retrospective	France (2014- 2020)	467	57 (19)	36	2/3	Fatty Liver	CT	LOS
Wu et al.	2019	Retrospective	China (2104–2016)	656	43.93 (9.81)	37	2/3	NAFLD	CT	Severity, Organ failure, SIRS
Yoon et al.	2017	Retrospective	South Korea (2009–2016)	200	54.3 (17.5)	40.5	2/3	Fatty Liver	CT Liver/spleen	Mortality, Severity, ANC, WON, AFC, PC, Organ failure, CRP
Zhang et al.	2021	Retrospective	China (2013–2020)	234	52.35 (17.64)	44	2/3	Fatty liver	N/A	Mortality
Xiao et al.	2012	Retrospective	China (2009–2011)	50	44 (12)	13.1	2/3	Fatty liver	MR	Severity
Jasdanwala et al.	2015	Retrospective	USA (N/A)	574	N/A	65.5	2/3	NAFLD	CT, US	Mortality, Severity, LOS
Simons-Linares, Marrero et al.^b^	2020	Retrospective	USA (2003–2013)	2,860,464	52.83 (N/A)	N/A	ICD	Cirrhosis	ICD	Mortality, Sepsis, Renal failure, SIRS
Simons-Linares, Suha et al.	2020	Retrospective	USA (2007–2017)	120	59,9 (N/A)	50	2/3	Cirrhosis	Biopsy or elastography or cross-sectional imaging	Mortality, Severity, ANC, AFC, PC, Systemic complication, SIRS, Renal, Cardiac, Respiratory failure
Vogel et al.	2022	Retrospective	Germany (2011–2020)	156	N/A	28.2	2/3	Cirrhosis	Fibrsocan, Clinical laboratory findings imagining	ANC, AFC, PC, Organ Failure
Jing et al.	2018	Retrospective	China (2016–2017)	128	52.7 (14.9)	40.6	2/3	Fatty liver	CT Liver/spleen	Severity, ANC, AFC, Organ Failure
Morel-Cerda et al.	2019	Retrospective	Spain (2017–2018)	186	41 (17.7)	63	2/3	Fatty liver	USG	Severity, Organ Failure, SIRS
Dou et al.	2017	Retrospective	China (2013–2016)	251	45.37 (15.46)	N/A	2/3	NAFLD	USG	Severity, LOS
Yan et al.	2020	Retrospective	China (2019–2019)	398	48 (N/A)	39.3	2/3	NAFLD	USG, CT	Severity, ANC, WON, AFC, PC, Organ Failure, MODS, SIRS, LOS, CRP
Váncsa et al.	Preprint	Prospective	Hungary (2013–2019)	2053	57 (17)	43.9	2/3	MAFLD	imaging + obesity or DM2, or metabolic dysregulation	Mortality, ANC, AFC, PC, Local, Systematic complication, Renal, Respiratory, Cardiac Failure
Ze-Hua et al.	2012	Retrospective	China (2010–2011)	606	50 (16)	43.7	2/3	Fatty liver	CT Liver/spleen	Mortality
Simons-Linares CR et al.^b,c^	2019	Retrospective	USA (2010–2015)	1,575,148	52.485 (17.22)	N/A	ICD	Cirrhosis	ICD9	Mortality, Infections, Sepsis, Renal Failure
Jimenez et al.^c^	2021	Prospective	Spain (2017–2017)	208	65.4 (N/A)	51	2/3	Cirrhosis	N/A	Severity, Local complication
Mahfouz et al.^b,c^	2022	Retrospective	USA (2016–2019)	74,095	N/A	N/A	ICD	Cirrhosis	ICD10	Mortality, LOS
Lupulescu et al.^c^	2019	Retrospective	Romania	150	55.9 (16.4)	42	N/A	Fatty liver	USG	Severity
Evans et al.^c^	2019	Retrospective	USA (2016–2018)	844	49 (N/A)	N/A	2/3	Cirrhosis	N/A	Mortality, LOS
Simons-Linares, Chittajallu et al.^b,c^	2019	Retrospective	USA (2009–2019)	409	N/A	N/A	N/A	Cirrhosis	N/A	IPN
Wang et al.^c^	2013	Retrospective	China (2010–2011)	120	N/A	N/A	N/A	Fatty liver	N/A	Severity, Organ failure, Systematic complication, SIRS, Respiratory failure
Hao et al.^c^	2015	Retrospective	China (2011–2013)	148	N/A	N/A	N/A	Fatty liver	N/A	Severity, Systemic complications, LOS
Shah et al.^c^	2020	Retrospective	USA (2010–2014)	2,147,200	51.36 (N/A)	N/A	ICD	NAFLD	ICD	Mortality, PC, Infections, SIRS, Respiratory failure, LOS
Make et al.^c^	2022	N/A	India (Ongoing study)	58	N/A	N/A	N/A	Fatty liver	CT Liver/spleen	Mortality, Severity, Local complication, Organ failure
Satapathy et al.^c^	2010	Retrospective	USA (2002–2009)	108	53 (N/A)	48	N/A	Fatty liver	CT Liver/spleen	ANC, AFC, LOS

AAP, alcohol-induced acute pancreatitis; AFC, acute peripancreatic fluid collections; AFLD, Alcoholic fatty liver disease; ANC, Acute necrotic collections; CLD, chronic liver disease; CRP, C-reactive protein; CT, computed tomography; CT Liver/spleen, ratio of density of liver to spleen; ICD, The International Classification of Diseases; IPN, Infectious pancreatic necrosis; LOS, Length of hospital stay; MAFLD, Metabolic associated fatty liver disease; MRI, Magnetic resonance imaging; NA, not available; NAFLD, Non-alcoholic fatty liver disease; ODS, Multiple Organ Dysfunction Syndrome; PC, Pancreatic pseudocysts; SD, standard deviation; SIRS, Systemic Inflammatory Response Syndrome; US, Ultrasonography; WON, Walled-off necrosis.

^a^Parameters represented as mean with standard deviation, or median with range (minimum and maximum).

^b^Study included only in systematic review.

^c^Conference abstract.

### Mortality and severity of AP in patients with CLD

**Figure 2:**
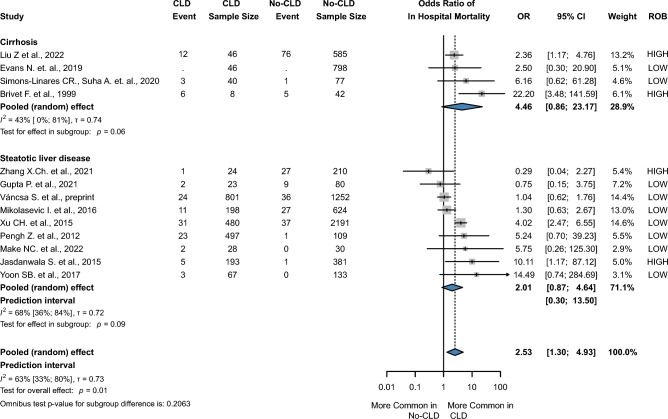
Forest plot of the pooled odds ratio of severe acute pancreatitis. OR, odds ratio; CI, confidence interval; CIL, lower limit of confidence interval; CIU, upper limit of confidence interval; CLD, chronic liver diseases.

**Figure 3:**
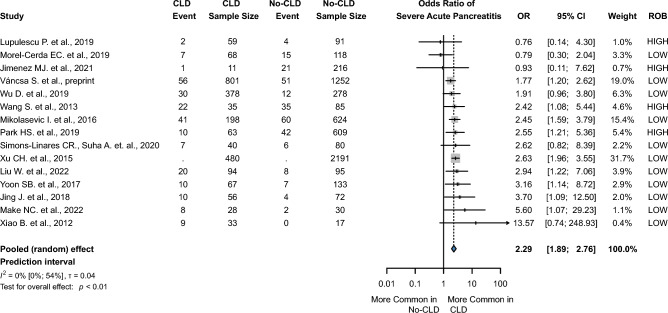
Forest plot of the pooled odds ratio of in-hospital mortality. OR, odds ratio; CI, confidence interval; CIL, lower limit of confidence interval; CIU, upper limit of confidence interval; CLD, chronic liver diseases.

A total of 13 publications^11,12,14,16,19,22,24,25,31,39,40,46,49^ reported mortality. CLD in patients with AP significantly increased the odds of in-hospital mortality with an OR of 2.53 (CI 1.30 to 4.93, I^2^ = 63%, *p* = 0.01), which was a low level of evidence according to GRADEpro. In the subgroup analysis, the pooled OR for in-hospital mortality for in-patients with SLD was 2.01 (CI 0.87 to 4.64, I^2^ = 66%, *p* = 0.09) compared to OR 4.46 (CI 0.86 to 23.17, I^2^ = 43%, *p* = 0.06) in the cirrhotic group (Fig. 2). In addition, concomitant CLD was associated with a more severe clinical course of the disease. The odds ratio for severe AP was (2.29 CI1.89 to 2.76, I^2^ = 0%, *p* < 0.01), and the odds ratio for moderately severe AP was (2.07 CI 1.44 to 2.96, I^2^ = 69%, *p* = 0.002). (Fig. 3 and Supplementary Fig. S1).

### The systemic complications of AP in patients with CLD

**Figure 4:**
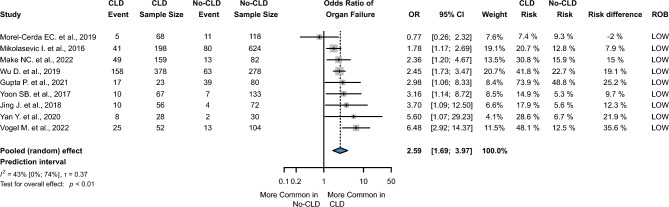
Forest plot of the pooled odds ratio for organ failure. OR, odds ratio; CI, confidence interval; CLD, chronic liver diseases.

**Figure 5:**
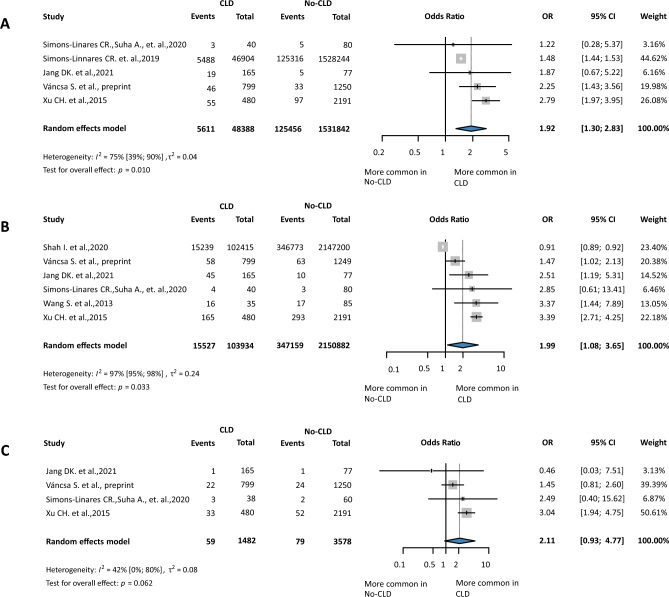
Forest plot of the pooled odds ratio for organ failure by organ type. (**A**) OR for renal failure; (**B**) OR for respiratory failure; (**C**) OR for cardiac failure. OR, odds ratio; CI, confidence interval; CLD, chronic liver diseases.

On the basis of nine studies^11,16,24,25,35,37,42,43,45^ and 2550 subjects, the odds of single organ failure increased 2.59-fold in patients with CLD (OR 2.59, CI1.69 to 3.97, I^2^ = 43%, *p* < 0.01). The number of Multiple Organ Dysfunction Syndrome (MODS) was reported in four studies^15,16,25,45^; CLD increased the odds 1.37-fold (OR 1.37, CI 1.07 to 1.75, I^2^ = 0%, *p* = 0.028), a very low level of evidence according to GRADEpro. (Fig. 4 and Supplementary Fig. S2). Furthermore, the analysis found that CLD may be associated with higher odds of renal, cardiac, and respiratory failure with pooled ORs of 1.92 (CI 1.30 to 2.83, I^2^ = 75%, *p* = 0.01), 2.11 (CI 0.93 to 4.77, I^2^ = 42%, *p* = 0.062), and 1.99 (CI 1.08 to 3.65, I^2^ = 97%, *p* = 0.033). (Fig. 5) SIRS was reported in the seven studies^21,31,36,37,39,43,45^ the OR were 1.95 (CI 1.03 to 3.68, I^2^ = 66%, *p* = 0.042). (Supplementary Fig. S4).

### Local complications of AP in patients with CLD

**Figure 6:**
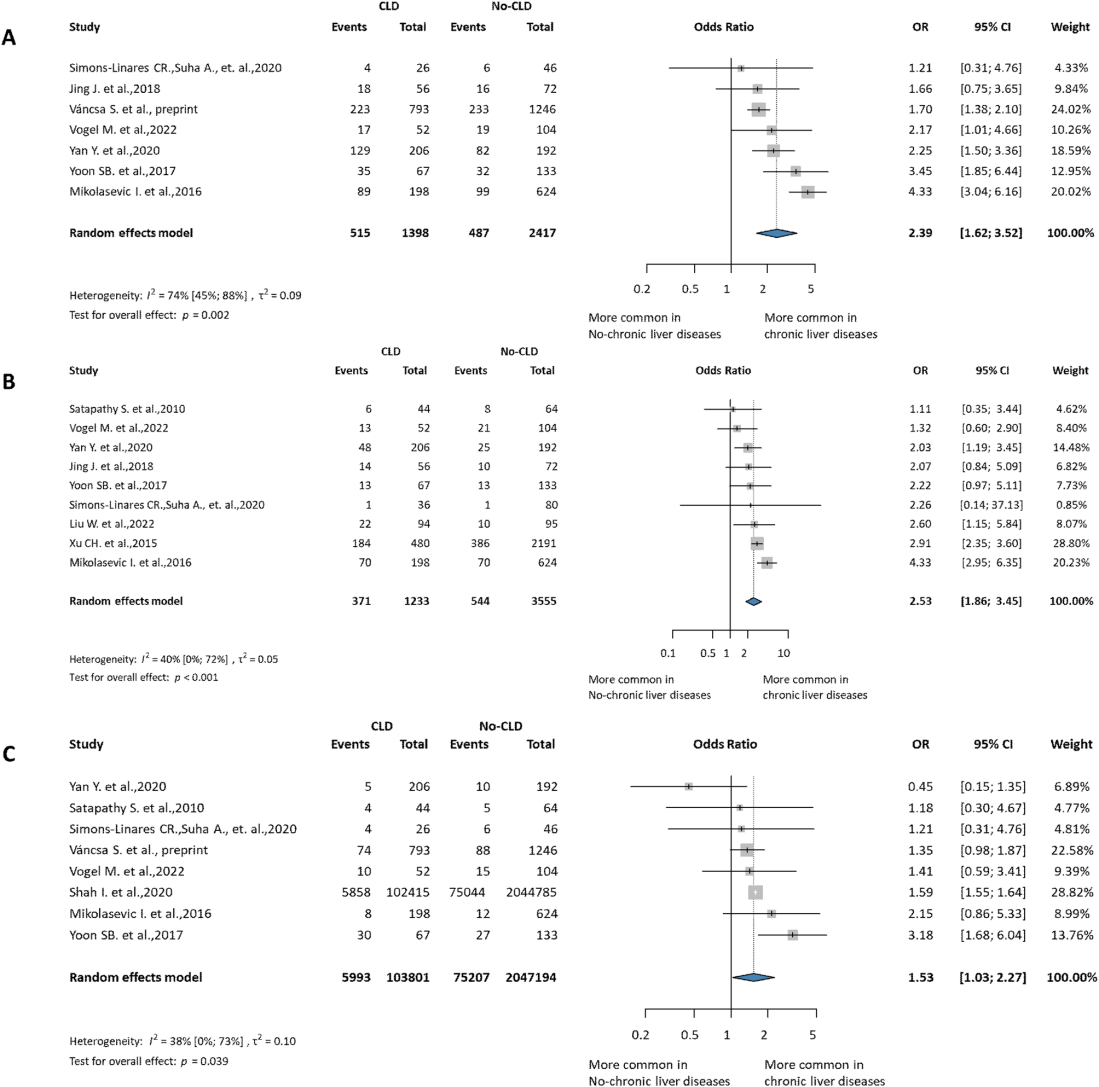
Forest plot of the pooled odds ratio of local complication. (**A**) OR for acute peripancreatic fluid collections. (**B**) OR for acute necrotic collection. (**C**) OR for pancreatic pseudocysts. OR, odds ratio; CI, confidence interval; CLD, chronic liver diseases.

CLD increased the odds of all measured local complications. Calculated from a total of nine publications^11,21,25,29,31,35,39,42,45^ with 4788 subjects, the OR for acute necrotic collections (ANC) was 2.53 times higher in the CLD group (CI 1.86 to 3.45, I^2^ = 40%, *p* = 0.001), a moderate level of evidence according to GRADEpro. Seven publications^11,25,31,35,42,45,49^ reported acute pancreatic fluid collection (APFC), (3815) with the pooled OR of 2.39 (1.62 to 3.52, I^2^ = 74%, *p* = 0.002), and eight publications reported pancreatic pseudocysts (PC)^11,25,29–31,35,45,49^ had an OR of 1.53 (1.03 to 2.27,I^2^ = 38% *p* = 0.039) (Fig. 6). For walled-off necrosis (WON), only two articles were found eligible^11,45^.

### Risk of bias assessment

**Figure 7:**
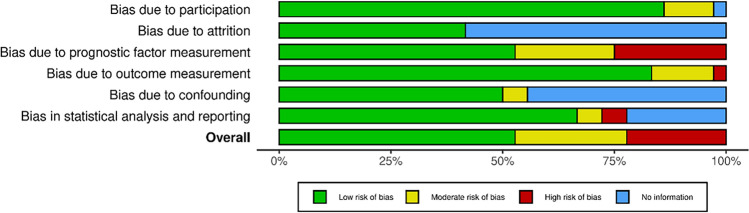
Summary plot of the risk of bias by the Robvis tool.

Half of the publications had a low ROB, 31% had a moderate, and 20% had a high ROB. This is the result of the high number of conference abstracts and poor diagnostic criteria for CLD in some studies. For an overview of ROB, see (Fig. 7) and Supplementary (Supplementary Table S1).

### Publication bias and heterogeneity

No sign of small study publication bias was detected in our analysis (Supplementary Figs. S5–9). However, some outcomes include less than ten studies, limiting the interpretation of small study publication bias.

## Discussion

This systematic review and meta-analysis investigated the impact of CLD on the outcomes among patients with AP. This work provides compelling evidence that CLD significantly increases both the mortality and severity of AP. Furthermore, our analysis showed that both local and systemic complications were higher in the CLD group.

Both CLD and AP are among the most common diseases in gastroenterology. The etiology of AP can be traced to biliary gallstones, which account for 45% of cases; alcohol consumption accounts for 20% of cases, AP caused by hypertriglyceridemia accounts for approximately 5% of cases, and the remaining 30% are due to less common causes^50^. Striking overlaps can be observed in the etiological factors of AP and CLD, such as the association between alcohol consumption and the risk of ALD or cirrhosis and alcohol-induced AP. Similarly, the relationship between the development of biliary gallstones or hypertriglyceridemia in obese patients with NAFDL/MAFLD overlaps with the risk of biliary and hypertriglyceridemia-induced AP in obese patients^51,52^.

This overlap is reflected in multiple studies that have demonstrated a higher incidence of CLD in patients with AP. For example, Simons-Linares et al. conducted a large database-based study of 2.8 million patients with AP in the US, which found a prevalence of cirrhosis of 2.8% (80,093), ten times higher than the prevalence of cirrhosis in the general US population (0.27%)^34,53^. Similarly, the Hungarian Pancreatic Study Group conducted a prospective cohort study and reported a 39% prevalence of MAFLD compared to a 23% prevalence in the general population. Furthermore, hypertriglyceridemia-induced AP was significantly more common in the MAFDL group (14% vs. 3%)^44,54^.

Our meta-analysis observed a tendency for worse outcomes for CLD patients in all measured parameters. The pooled OR for mortality was found to be 2.5 times higher in the CLD group, with statistical significance. Subgroup analysis of mortality showed a higher mortality trend in patients with cirrhosis. However, no definitive conclusion could be drawn due to the small sample size and broad CI. Multiple database-based studies with large sample sizes reported mortality and other outcomes, but these data could not be included in the meta-analysis due to overlapping populations^23,30,33,34^. However, the effect size in these studies was relatively similar to the pooled one, with mortality ORs for each study ranging from 1.8 to 2.4, increasing the validity of the meta-analysis findings. The database-based study with the largest sample by Simons-Linares et al.^34^ identified decompensated cirrhosis as the risk factor associated with the highest odds for in-hospital mortality, with an adjusted OR of 1.4 for decompensated vs. compensated cirrhosis and 2.4 for decompensated vs. no cirrhosis. CLD patients with decompensated cirrhosis constitute a specific subgroup with distinct comorbidities associated with liver malfunction. This makes these patients much more vulnerable, especially due to their susceptibility to trigger acute-on-chronic liver failure (ACLF), a syndrome characterized by acute decompensation of cirrhosis, organ failure(s), and high short-term mortality. Vogel et al.^35^ reported that the incidence of ACLF was 44% in AP patients with cirrhosis, with relatively high proportions of more severe grades II and III. This could be explained by the higher prevalence of organ failure in AP with CLD. A similar pattern could be observed in our data, where the pooled OR for single organ failure was 2.59 times higher and OR for multi-organ failure was 1.37 times higher in the CLD group. All these data suggest that the stage of CLD plays a crucial role in determining the prognosis of AP, despite the fact that the level of evidence is still very low.

Increased odds of local complications, including ANC, APFC, and PC, were observed. The odds of WON could not be determined due to insufficient data. The most interesting finding from a prognostic perspective is the 2.53-fold increase in the odds of ANC, as the presence of which increases mortality by up to 15%. If ANC becomes infected (IPN), mortality increases to 29%. Moreover, Simons-Linares et al.^32^ reported an OR of 2.45, *p* = 0.004 for infected pancreatic necrosis (IPN) in AP patients with cirrhosis^55^. The higher odds of local complications could contribute to worsening mortality in patients with CLD.

Furthermore, infections are also well-known common complications of CLD and could be another possible explanation for worse outcomes. Vogel et al.^35^ reported sepsis-associated MODS as a leading cause of death in their cohort, whereas several other authors reported an increased prevalence of infections^39,56^, need for antibiotics^29^, interventions due to infection^35^, infected pancreatic necrosis^32^, and the prevalence of sepsis^30,33,34^ in AP patients with CLD. The present meta-analysis also attempted to analyzed infectious complications, but due to large heterogeneity in definitions, overlapping populations, and unclear definitions, it was impossible to pool this data. Despite this, infections appear to play a significant role in AP patients with CLD, and the early use of antibiotics in these patients seems to be warranted.

The adverse effects of CLD on AP could be expedited by several factors. The most obvious one is the association of CLD with a number of comorbidities such as type 2 diabetes, arterial hypertension, obesity, cardiovascular disease, chronic kidney disease, and chronic obstructive lung disease^57–59^. Though, several authors^11,39^ propose an alternative explanation, namely, the presence of a chronic pro-inflammatory state in CLD, aggravating the severity of inflammation caused by AP^11^. This theory is supported by our finding that the odds of SIRS are doubled in the CLD group.

It remains uncertain if the effect of CLD is observed independently of the confounders. The analysis only with the adjusted models was attempted. However, due to the lack of these models in current literature, this became impossible. In the current literature, data on the adjusted models was found only for Mortality, Organ Failure, MODS, and SIRS. We used this data to perform a sensitivity analysis, where the adjusted model OR was pooled with the rest of the raw and not-adjusted OR values. For details, see the supplementary material (Supplementary Figs. S26–33). Overall, we found, that the effect sizes in the individual study level and the pooled effect sizes did not differ significantly between the adjusted and non-adjusted models. We did not detect any clinically meaningful difference between the adjusted and non-adjusted models. However, no definitive conclusion could be drawn due to the poor reporting of confounders in articles.

Throughout the results, relatively high level of clinical and statistical heterogeneity of the studies was observed. To address it, sensitivity analysis with a leave-one-out analysis was conducted. A few studies were found to be influential: affecting the pooled effect size, heterogeneity statistics, or other parameters in a relevant amount. Excluding these specific studies reduced heterogeneity in organ failure, SIRS, and APFC outcomes. For organ failure, the I^2^ value decreased from 43 to 8% by excluding the study by Vogel et al.^35^, likely because it was the only study using the Sequential Organ Failure Assessment classification (SOFA). In other cases, the reason remains unclear (Supplementary Figs. S16–25). No influential publication was detected for in-hospital mortality.

### Strengths and limitations

This meta-analysis has several strengths. Firstly, it employs large sample size and a diverse population from European, American, and Asian countries, making these data more applicable in practice. Furthermore, the study employs rigorous methodology with a pre-registered protocol and an assessment of the level of certainty by GRADEpro (Supplementary Table S3), which increases the reliability of the results.

However, several limitations must be noted. First, most of the data come from retrospective or cross-sectional studies, which limits the level of evidence. Second, the nature of the datasets reported in the current literature did not allow for more detailed subgrouping according to the specific etiology or stage of liver fibrosis. The additional limitation is the relatively high number of studies with moderate or high ROB. To address this, an analysis was conducted using only studies with a low risk of bias. This significantly changed heterogeneity only in the outcomes for systemic complications but did not affect other outcomes. The effect size remained the same (Supplementary Figs. S10–15).

### Implications for practice

These results imply that CLD is a significant risk factor for worse outcomes in AP. Physicians should be aware of this, especially in the early phase of AP, for proper risk stratification of these patients. AP patients with concomitant AP and CLD require more attention and rigorous monitoring during hospitalization, as they are at higher risk of more severe forms of diseases and of both local and systemic complications.

### Implications for research

The proclivity for simultaneous occurrence of AP and CLD and worse outcomes highlights the importance of further research into this relationship. Recent publications have shown that the rapid application of science to medical practice can dramatically improve healthcare, including reductions in duration and cost of hospitalizations and better patient outcomes^60^. Academia Europaea has suggested that the translational medicine cycle is one of the most effective strategies to achieve this goal^61^. Many unanswered questions about concomitant AP and CLD could benefit from this approach. One of the most pressing issues is the lack of prospective data, another is the exact role of infections and the possible role of prophylactic ATB and albumin substitutions. Inspiration could be drawn from well-established protocols for variceal bleeding and spontaneous bacterial peritonitis. Furthermore, CLD could be incorporated into future prognostic models of AP severity.

## Conclusion

Chronic liver disease is an important risk factor for unfavorable outcomes in patients with AP, leading to higher mortality and increased odds of local and systemic complications.

## Methods

The Cochrane Handbook was followed for standards and methods^62^, and PRISMA^63^ for reporting (Supplementary Table S2). The protocol was registered in advance on Prospero with code IDCRD42022368905.

### Eligibility criteria

Studies were included if they met the following criteria: the study reported on a population of adult patients diagnosed with AP, where patients exposed to CLD were compared to control patients without records or signs of CLD. The primary outcome was mortality; secondary outcomes were severity of AP, local or systemic complications, and the length of hospital stay. CLD was defined as liver disease lasting more than six months. Randomized control trials, retrospective and prospective cohorts were included, while case reports or series were excluded.

### Information sources

On 10 October 2022, a systematic search was conducted with the same search key and without language or other restrictions, using: Embase, Medline, and Central. The references of studies included were systematically searched using citation chaser^47^.

### Search strategy

The search key is included in Supplementary Document 1.

### Selection process

Screening and selection were performed entirely by two independent review authors (JH and RB) in a stepwise manner. Automatic and manual duplicate removal was performed in EndNote X20^64^. Selection by title and abstract was performed in Rayyan^65^. The level of agreement was assessed at each step by Cohen’s kappa coefficient. In case of disagreement during the selection, a third independent party was assigned to resolve it.

### Data collection process

Data extraction was performed from the eligible articles by two independent authors (JH and RB). A predefined Excel table was used for data collection. To minimize the chance of error, data collections were performed separately with subsequent comparisons of extracted data. A third independent party (PJH) resolved all disagreements.

### Data items

The following data were extracted: first author, year of publication, study population size, study location and period, AP diagnosis criteria, CLD type and diagnostic criteria, mortality rate, severity of AP, number of local complications such as ANC, APFC, PC, WON, number of systematic complications such as SIRS, number of infections, sepsis, respiratory, renal and cardiac failure, and length of stay.

### Study risk of bias assessment (ROB)

The QUIPS^66^ tool was used for ROB by two independent investigators (JH and RB). Any disagreement was resolved by an independent third party. The results were visualized using the robvis tool^67^.

### The level of evidence

The level of evidence was assessed according to the GRADEpro tool.

### Synthesis methods

As we assumed considerable between-study heterogeneity at all cases, therefore a random-effects model was used to pool effect sizes in a frequentist framework.

To calculate the study odds ratios (OR) and the pooled OR, the total number of patients and those with the event of interest (referred as “raw data”) in each group separately were extracted or calculated from the studies where available. We reported the results as the odds of event of interest in experimental group versus the odds of event of interest in the control group. In cases where OR was given without “raw data”, we used the OR and its 95% confidence interval (CI) (hereafter referred as “direct OR” data). For estimating the pooled value, the Mantel–Haenszel (for “raw data”) and the inverse variance method (for situations where “direct OR” was present) were used.

Results were considered statistically significant if the pooled 95% CI did not contain the null value (*p* < 5%). Where applicable—the number of low-risk studies was larger than 5-, we reported the prediction intervals (i.e. the expected range of effects of future studies) of results too. In order to better understand the clinical effect sizes, the risks of the outcomes and their differences between the two groups for each study are shown in the graphs too (in case of “raw data”).

All statistical analyses were performed with R^68^ (v4.3.3) using the meta^69^ (v7.0.0) package for basic meta-analysis calculations and plots, and dmetar^70^ (v7.0.0) package for additional influential analysis calculations and plots.

Small study publication bias was assessed by visual inspection of funnel-plots and calculating Egger’s or Harbord (for “raw data”) test *p* values^71^. We assumed possible small study bias if the p-value was less than 10%. (Although we kept in mind that the diagnostic assessment of this test was limited, below ~ 10 studies.)

Additional details are provided in Supplementary Document 2.

### Ethical approval

No ethical approval was required for this systematic review with meta-analysis, as all data were already published. No patients were involved in the design, conduct, or interpretation of our study.

### Supplementary Information

Supplementary Information.

## Data Availability

The datasets used in this study can be found in the full-text articles included in this systematic review and meta-analysis.
