# Supplementary material for: Chronic liver disease is an important risk factor for worse outcomes in acute pancreatitis: a systematic review and meta-analysis
Source: Sci Rep. 2024 Jul 19;14:16723. doi: 10.1038/s41598-024-66710-w (PMC11271551; doi:10.1038/s41598-024-66710-w)
Supplement: Supplementary file 1 — Supplementary Information. [file 41598_2024_66710_MOESM1_ESM.pdf]

Title

Chronic liver disease is an important risk factor for worse outcomes in acute pancreatitis: a systematic review and meta-analysis

Authors

Jakub Hoferica[1,2], Ruben Zsolt Borbély[1,3], Ali Nedjati Aghdam[1], Eszter Ágnes Szalai[1,4], Ádám Zolcsák[1,5], Dániel Sándor Veres[1,5], Bálint Erőss[1,7], Krisztina Hagymási[1,6], Péter Hegyi [1,7,8,9], Peter Banovcin[1,2]†, Péter Jenő Hegyi[1,7]†*

Affiliations:

[1] Centre for Translational Medicine, Semmelweis University, Budapest, Hungary
[2] Jessenius Faculty of Medicine in Martin, Comenius University, Martin, Slovakia
[3] Department of Medical Imaging, Bajcsy-Zsilinszky Hospital and Clinic, Budapest, Hungary
[4] Department of Restorative Dentistry and Endodontics, Semmelweis University, Budapest, Hungary
[5] Department of Biophysics and Radiation Biology, Semmelweis University, Budapest, Hungary
[6] Department of Surgery, Transplantation and Gastroenterology, Semmelweis University, Budapest, Hungary
[7] Institute of Pancreatic Diseases, Semmelweis University, Budapest, Hungary
[8] Institute for Translational Medicine, Medical School, University of Pécs, Pécs, Hungary.
[9] Translational Pancreatology Research Group, Interdisciplinary Centre of Excellence for Research Development and Innovation University of Szeged, Szeged, Hungary

† These two authors contributed equally

*** Corresponding author**

Péter Jenő Hegyi MD, PHD, MPH, MHA

Institute of Pancreatic Diseases, Semmelweis University, Budapest, Hungary

Postal address: H-1083 Budapest, Tömő utca 25-29, Hungary

Tel.: +36 70 387 0157

E-mail address: hegyi.peter1@semmelweis-univ.hu

Supplementary materials

Document 1: Search key

Document 2: Detailed effect measure and synthesis methods

Figures in supplementary materials

Figure S1: Forest plot of the pooled odds ratio of moderately severe acute pancreatitis

OR: odds ratio; CI: confidence interval; CLD: chronic liver diseases

Figure S2: Forest plot of the pooled odds ratio of mild acute pancreatitis

OR: odds ratio; CI: confidence interval; CLD: chronic liver diseases

Figure S3: Forest plot of the pooled odds ratio for multiorgan failure

OR: odds ratio; CI: confidence interval; CLD: chronic liver diseases

Figure S4: Forest plot of the pooled odds ratio for Systemic Inflammatory Response

OR: odds ratio; CI: confidence interval; CLD: chronic liver diseases

Figure S5: Contour-enhanced funnel plot of the pooled odds ratio of in-hospital mortality

Figure S6: Contour-enhanced funnel plot of the pooled odds ratio of severe acute pancreatitis

Figure S7: Contour-enhanced funnel plot of the pooled odds ratio of moderately severe acute pancreatitis

Figure S8: Contour-enhanced funnel plot of the pooled odds ratio mild acute pancreatitis

Figure S9: Contour-enhanced funnel plot of the pooled odds ratio of acute necrotic collection

Figure S10: The OR for the Systemic Inflammatory Response (SIRS) in patients with AP and with and without CLD in the studies with low ROB.

OR: odds ratio; CI: confidence interval; CLD: chronic liver diseases

Figure S11: The OR for Severe Acute Pancreatitis (SAP) in patients with AP and with and without CLD in the studies with low ROB.

OR: odds ratio; CI: confidence interval; CLD: chronic liver diseases

Figure S12: The OR for In-Hospital Mortality in patients with AP and with and without CLD in the studies with low ROB.

OR: odds ratio; CI: confidence interval; CLD: chronic liver diseases

Figure S13: The OR for Organ Failure in patients with AP and with and without CLD in the studies with low ROB.

OR: odds ratio; CI: confidence interval; CLD: chronic liver diseases

Figure S14: The OR for Systemic complications in patients with AP and with and without CLD in the studies with low ROB.

A: OR for renal failure; B: OR for respiratory failure; C: OR for cardiac failure.
OR: odds ratio; CI: confidence interval; CLD: chronic liver diseases

Figure S15: The OR for Local complications in patients with AP and with and without CLD in the studies with low ROB

A: OR for acute peripancreatic fluid collections B: OR for acute necrotic collection C: OR for pancreatic pseudocysts.
OR: odds ratio; CI: confidence interval; CLD: chronic liver diseases

Figure S16: The leave-one-out analysis for the Systemic Inflammatory Response

CI: confidence interval; CLD: chronic liver diseases

Figure S17: The leave-one-out analysis for Severe Acute Pancreatitis

CI: confidence interval; CLD: chronic liver diseases

Figure S18: The leave-one-out analysis for In-Hospital Mortality

CI: confidence interval; CLD: chronic liver diseases

Figure S19: The leave-one-out analysis for Organ Failure in patients

CI: confidence interval; CLD: chronic liver diseases

Figure S20: The leave-one-out analysis for Cardiac Failure

CI: confidence interval; CLD: chronic liver diseases

Figure S21: The leave-one-out analysis for Respiratory Failure

CI: confidence interval; CLD: chronic liver diseases

Figure S22: The leave-one-out analysis for Renal Failure

CI: confidence interval; CLD: chronic liver diseases

Figure S23: The leave-one-out analysis for Acute Necrotic Collection

CI: confidence interval; CLD: chronic liver diseases

Figure S24: The leave-one-out analysis for Pancreatic Pseudocysts

CI: confidence interval; CLD: chronic liver diseases

Figure S25: The leave-one-out analysis for Acute Peripancreatic Fluid Collections

CI: confidence interval; CLD: chronic liver diseases

Figure S26: The analysis, including adjusted models for SIRS

OR: odds ratio; CI: confidence interval; CLD: chronic liver diseases

Figure S27: The leave-one-out analysis including adjusted models for SIRS

CI: confidence interval; CLD: chronic liver diseases

Figure S28: The analysis, including adjusted models for Organ Failure

OR: odds ratio; CI: confidence interval; CLD: chronic liver diseases

Figure S29: The leave-one-out analysis analysis, including adjusted models for Organ Failure

CI: confidence interval; CLD: chronic liver diseases

Figure S30: The analysis, including adjusted models for Severe Acute Pancreatitis

OR: odds ratio; CI: confidence interval; CLD: chronic liver diseases

Figure S31: The leave-one-out analysis, including adjusted models for Severe Acute Pancreatitis

CI: confidence interval; CLD: chronic liver diseases

Figure S32: The analysis, including adjusted models for Severe Acute Pancreatitis

OR: odds ratio; CI: confidence interval; CLD: chronic liver diseases

Figure S33: The leave-one-out analysis, including adjusted models for Severe Acute Pancreatitis

CI: confidence interval; CLD: chronic liver diseases

Tables in supplementary materials

Table S1: Traffic light plot of the risk of bias

Table S2: PRISMA checklist

Table S3: GRADEpro assessment of the level of evidence

OR: odds ratio; CI: confidence interval; CLD: chronic liver diseases

References for supplementary materials

Document 1: Search key

("Acute pancreatitis" OR "biliary pancreatitis") AND ((("liver" or "hepat*") AND (disease or failure or insufficiency OR Fatty)) OR cirrhosis OR 'sclerosing cholangitis' OR ' biliary cholangitis' OR MAFLD OR NAFLD OR steatosis OR steatohepatitis OR NASH OR antitrypsin OR "Wilson disease" OR "hemochromatosis" OR "hepatitis").

There was one deviation from the protocol regarding the search key where we extended it to include (biliary pancreatitis).

Document 2: Detailed effect measure and synthesis methods

We provide the following additional details on data synthesis:

As we assumed considerable between-study heterogeneity at all cases, therefore a random-effects model was used to pool effect sizes in a frequentist framework.

To calculate the the study odds ratios (OR) and the pooled OR, the total number of patients and those with the event of interest (hereafter referred as "raw data") in each group separately were extracted or calculated from the studies where available. We reported the results as the odds of event of interest in experimental group versus the odds of event of interest in the control group. In cases where OR was given without "raw data", we used the OR and its 95% confidence interval (CI) (hereafter referred as "direct OR" data).

Pooled OR based on "raw data" was calculated by the Mantel-Haenszel method[1,2]. Exact Mantel-Haenszel method (without continuity correction) was used to handle zero cell counts (as recommended[3,4]) for "raw data". Inverse variance weighting method was used to calculate the pooled OR (based on directly given OR without "raw data"). In these cases zero cell counts were handled by adding 0.5 as continuity correction. We used a Hartung-Knapp adjustment[5,6]] for CIs. This adjustment was applied only if it is more conservative then the classical one (as recommended in Jackson et al.[7] as hybrid method 2).

To estimate the heterogeneity variance measure (τ2), for "raw data" OR calculation the Paule-Mandel method [8]and for "direct" OR calculation the restricted maximum-likelihood estimator was used with the Q profile method for confidence interval (recommended by Veroniki et al.[9] and Harrer et al. [10]).

Where applicable - the number of low risk studies were larger than 5 -, we reported the prediction intervals (i.e. the expected range of effects of future studies) of results too. Prediction interval calculations were based on t-distribution.

Additionally, between-study heterogeneity was described by the Higgins&Thompson's I2 statistics[10].

In order to better understand the clinical effect sizes, the risks of the outcomes and their differences between the two groups for each study are shown in the graphs too (in case of "raw data"). We reported our results on forest plots. In case of 0 cell counts, individual study OR with 95% CI was calculated by adding 0.5 as continuity correction. Results were considered statistically significant if the pooled 95% CI did not contain the null value ($p < 5\%$).

Small study publication bias was assessed by visual inspection of funnel-plots and calculating Egger's or Harbord (for "raw data") test p-values[11]. We assumed possible small study bias if the p-value was less than 10%. (Although we kept in mind that the diagnostic assessment of this test was limited, below ~10 studies.)

In case of subgroup analysis we used a fixed-effects "plural" model (aka. mixed-effects model). We assumed different $\tau 2$ values in the subgroups. To asses the difference between the subgroups an "omnibus Cochran's Q" test was used between subgroups[10]. The null hypothesis was rejected on a 5% significance level. The subgroup analysis was planned before the data extraction in the case of the stage of CLD variables.

Potential outlier publications was explored using different leave-one-out influence measures and plots following the recommendation of Harrer et. al[10]. The short description of these parameters are given in the caption of the figures. We made subset analyses for studies were the risk of bias was low too.

We performed extra analyses to reveal some potential biases: with including adjusted odds ratios (in comparison with adjusted values). For these analyses the methodology was the same as in case of "direct OR" values.

Figures in supplementary materials

Figure S1: Forest plot of the pooled odds ratio of moderately severe acute pancreatitis

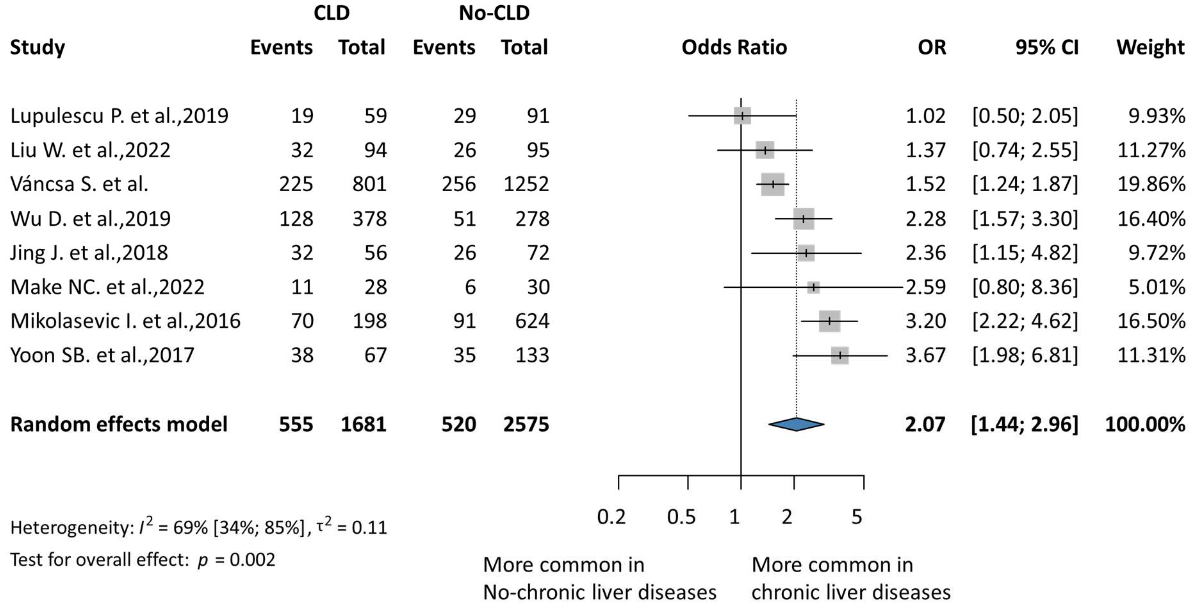

Figure S2: Forest plot of the pooled odds ratio of mild acute pancreatitis

	CLD		No-CLD				
Study	Events	Total	Events	Total	OR	95% CI	Weight
Xiao B. et al.,2012	24	33	17	17	0.07	[0.00; 1.35]	2.11%
Jasdanwala S. et al.,2015	109	193	351	381	0.11	[0.07; 0.18]	9.11%
Jang DK. et al.,2021	90	187	47	55	0.16	[0.07; 0.35]	7.78%
Make NC. et al.,2022	9	28	22	30	0.17	[0.06; 0.53]	6.38%
Yoon SB. et al.,2017	19	67	91	133	0.18	[0.10; 0.35]	8.45%
Jing J. et al.,2018	14	56	42	72	0.24	[0.11; 0.51]	7.94%
Mikolasevic I. et al.,2016	86	198	472	624	0.25	[0.18; 0.35]	9.53%
Wu D. et al.,2019	220	378	215	278	0.41	[0.29; 0.58]	9.50%
Liu W. et al.,2022	42	94	61	95	0.45	[0.25; 0.81]	8.69%
Váncsa S. et al.	520	801	945	1252	0.60	[0.50; 0.73]	9.84%
Lupulescu P. et al.,2019	38	59	58	91	1.03	[0.52; 2.04]	8.29%
Jimenez MJ. et al., 2021	10	11	195	216	1.08	[0.13; 8.83]	3.38%
Dou J. et al.,2017	68	117	61	134	1.66	[1.01; 2.74]	9.00%
**Random effects model**	**1249**	**2222**	**2577**	**3378**	**0.35**	**[0.21; 0.59]**	**100.00%**

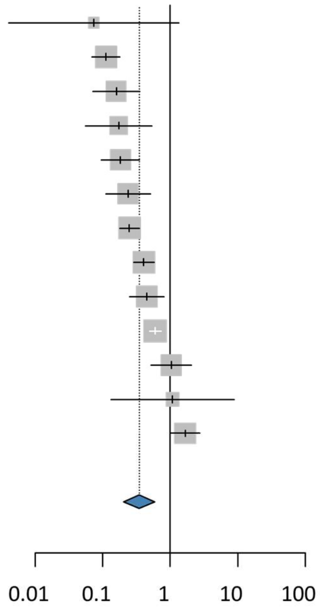

Heterogeneity: $I^2 = 89\%$ [83%; 93%], $\tau^2 = 0.59$
Test for overall effect: $p < 0.001$

Figure S3: Forest plot of the pooled odds ratio for multiorgan failure

	chronic liver diseases		No-chronic liver diseases							
Study	Events	Total	Events	Total	OR	CIL	CIU	OR	95% CI	Weight
Frey C. et al.,2007	.	4649	.	80064	1.30	1.10	1.60	1.30	[1.10; 1.60]	84.14%
Yan Y. et al.,2020	26	206	17	192				1.49	[0.78; 2.84]	7.08%
Gupta P. et al.,2021	3	23	7	80				1.56	[0.37; 6.60]	1.42%
Mikolasevic I. et al.,2016	17	198	26	624				2.16	[1.15; 4.07]	7.36%
**Random effects model (HK)**		**5076**		**80960**				**1.37**	**[1.07; 1.75]**	**100.00%**

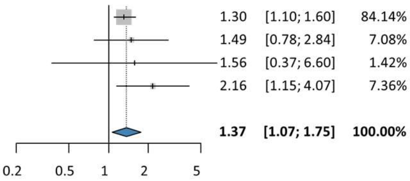

Heterogeneity: $I^2 = 0\%$ [0%; 85%] $\tau^2 = 0$
Test for overall effect: $p = 0.028$

Figure S4: Forest plot of the pooled odds ratio for Systemic Inflammatory Response

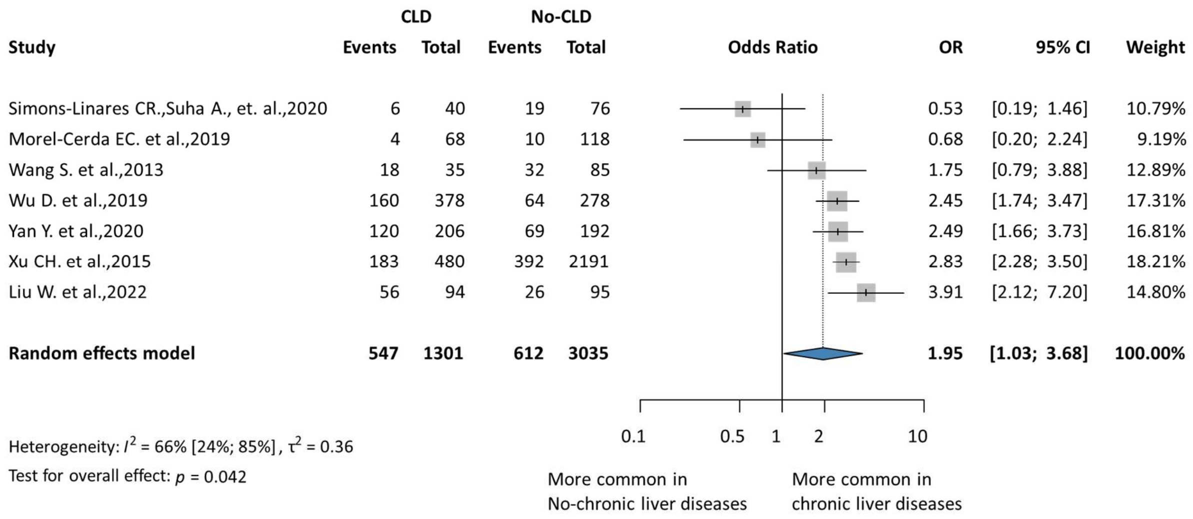

Figure S5: Contour-enhanced funnel plot of the pooled odds ratio of in-hospital mortality

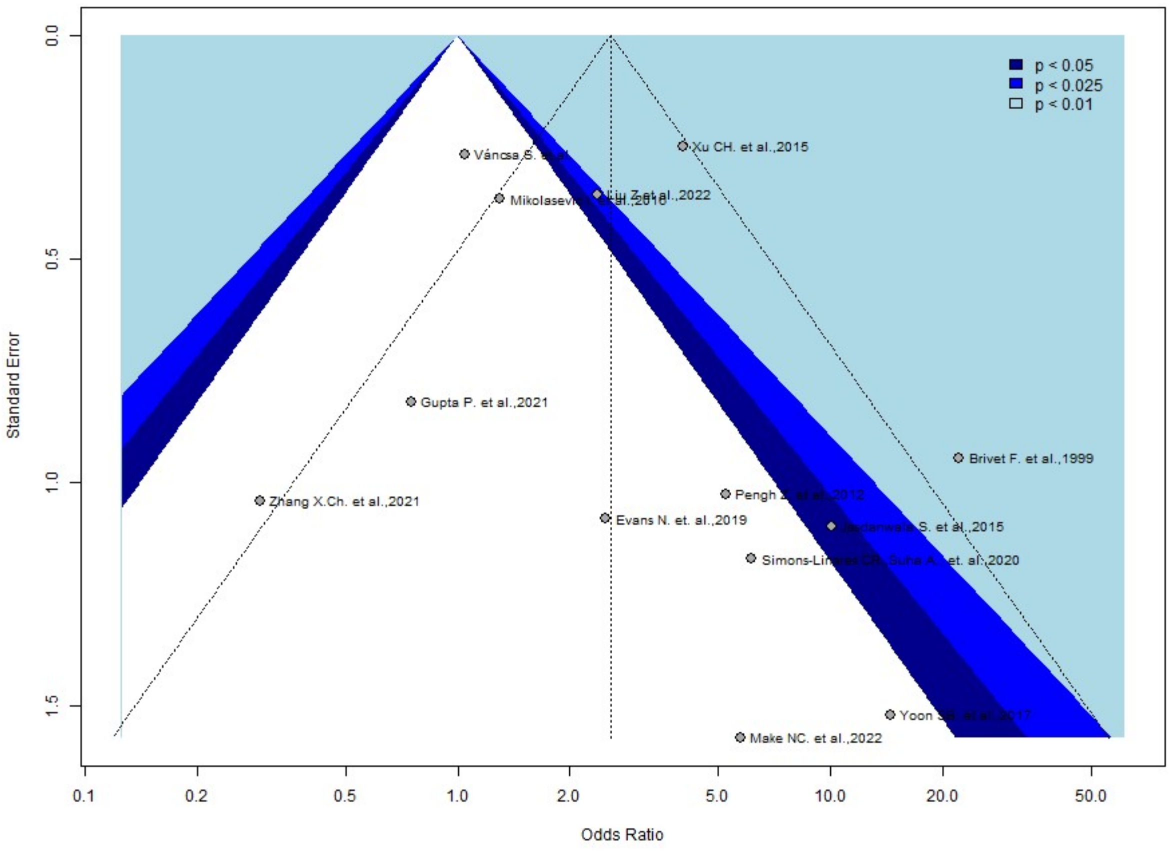

Figure S6: Contour-enhanced funnel plot of the pooled odds ratio of severe acute pancreatitis

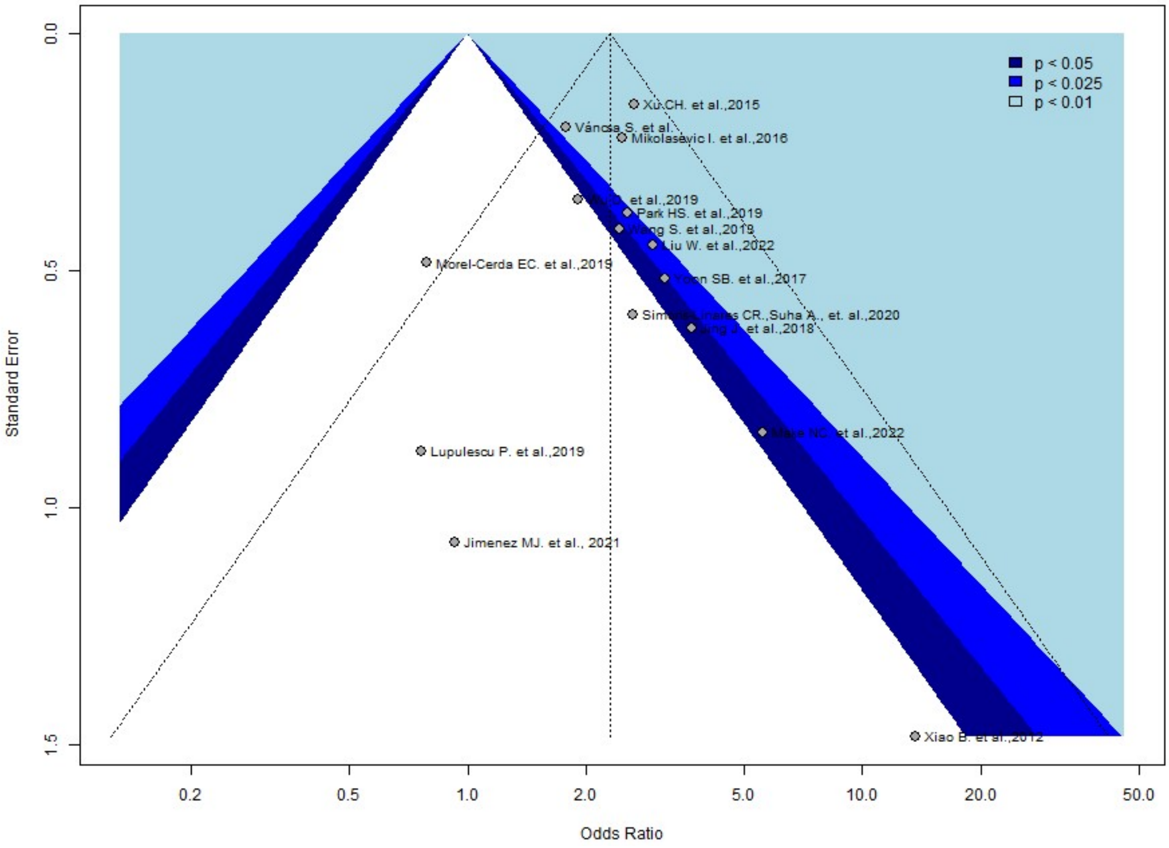

Figure S7: Contour-enhanced funnel plot of the pooled odds ratio of moderately severe acute pancreatitis

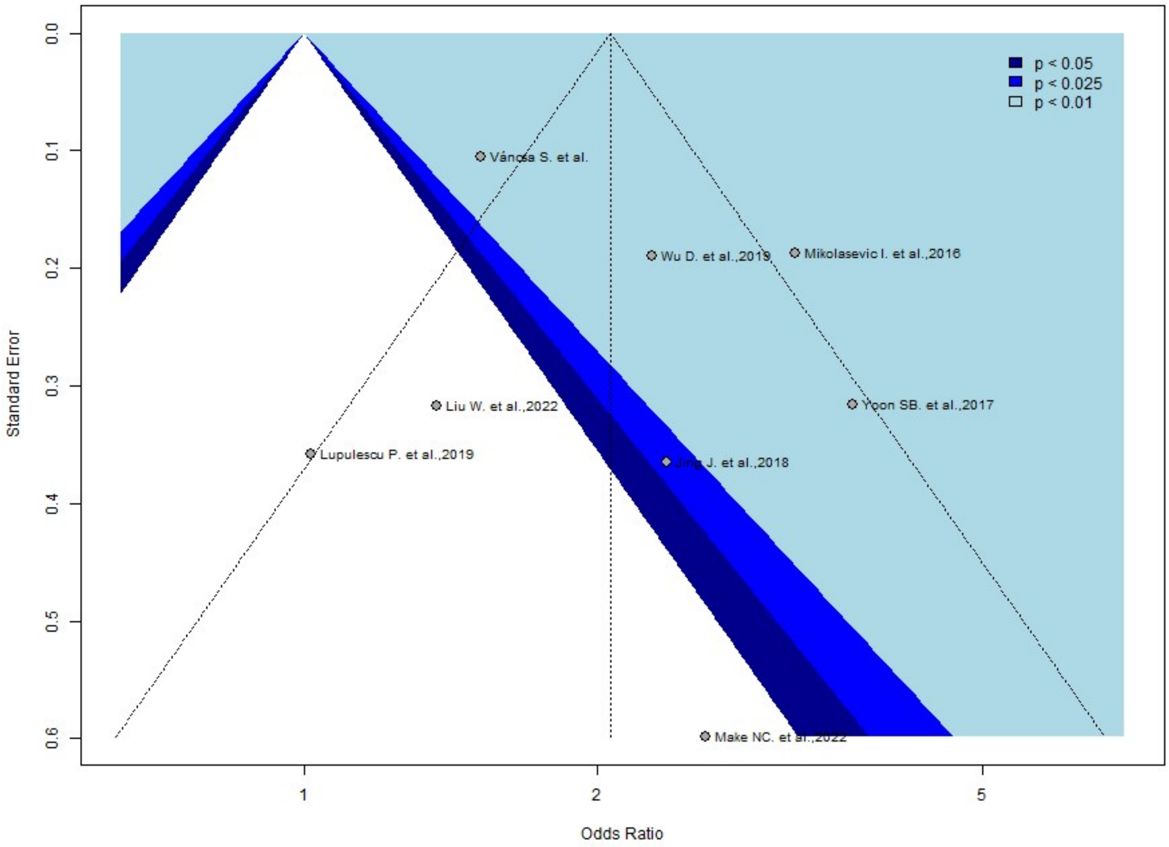

Figure S8: Contour-enhanced funnel plot of the pooled odds ratio of mild acute pancreatitis

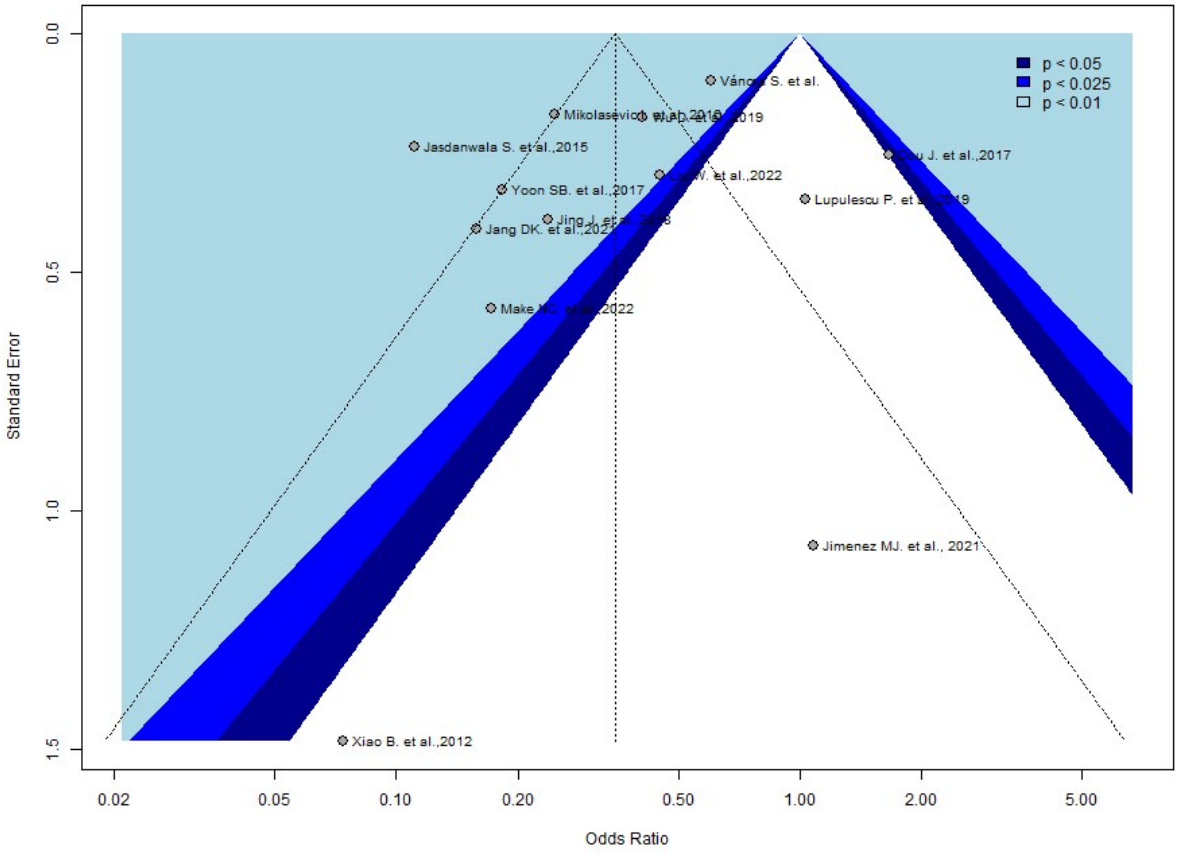

Figure S9: Contour-enhanced funnel plot of the pooled odds ratio of local complication B for acute necrotic collection

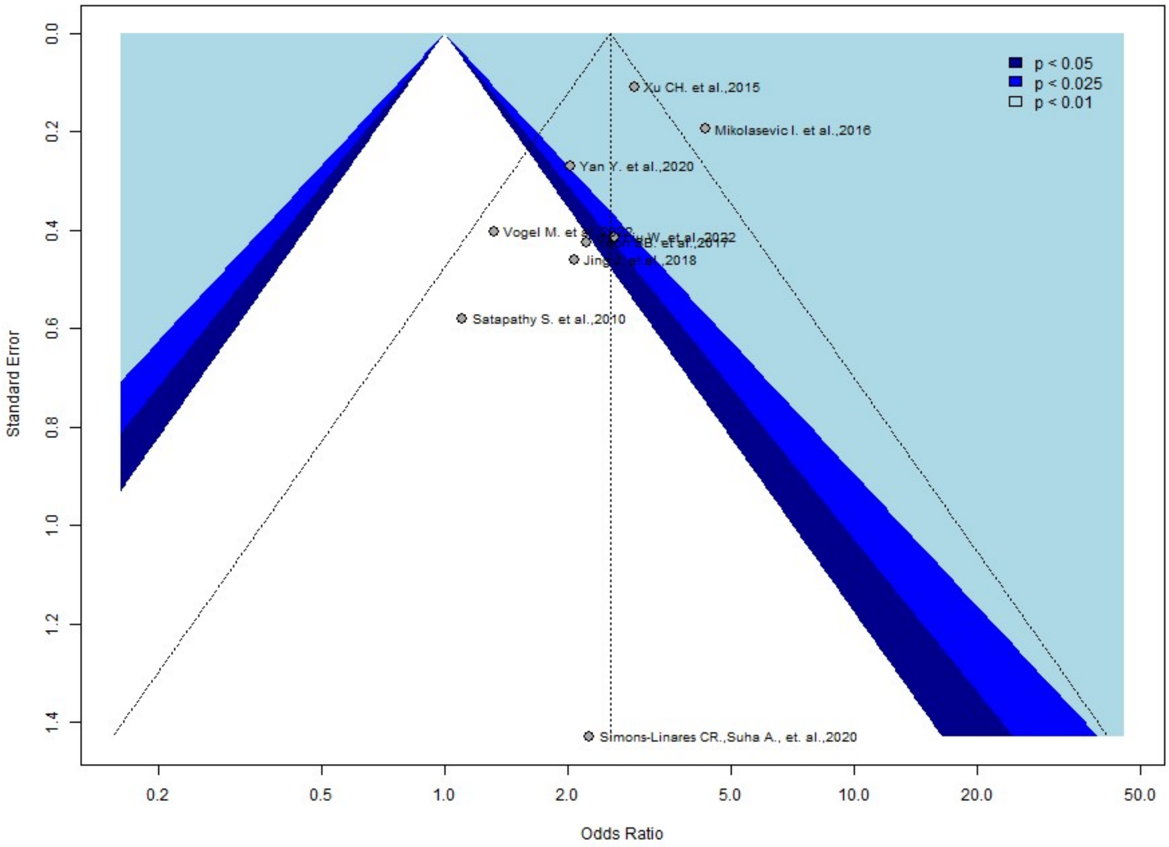

Figure S10: The OR for the Systemic Inflammatory Response (SIRS) in patients with AP and with and without CLD in the studies with low ROB.

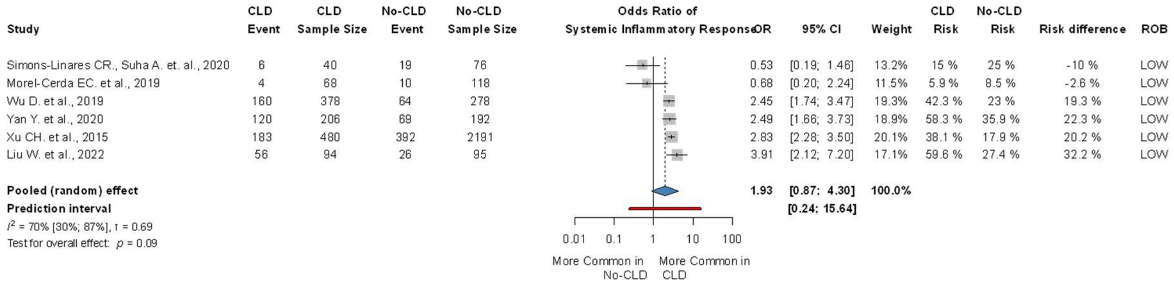

Figure S11: The OR for Severe Acute Pancreatitis (SAP) in patients with AP and with and without CLD in the studies with low ROB.

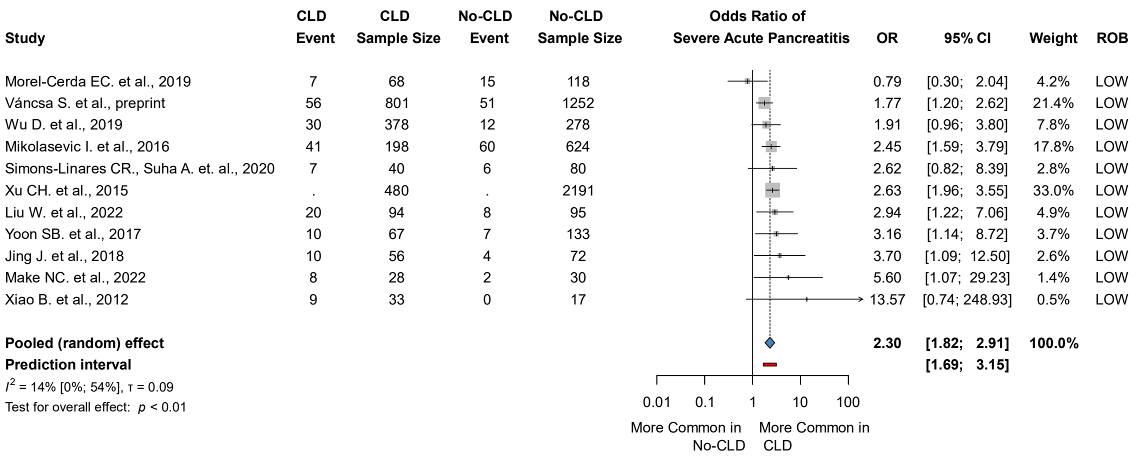

Figure S12: The OR for In-Hospital Mortality in patients with AP and with and without CLD in the studies with low ROB

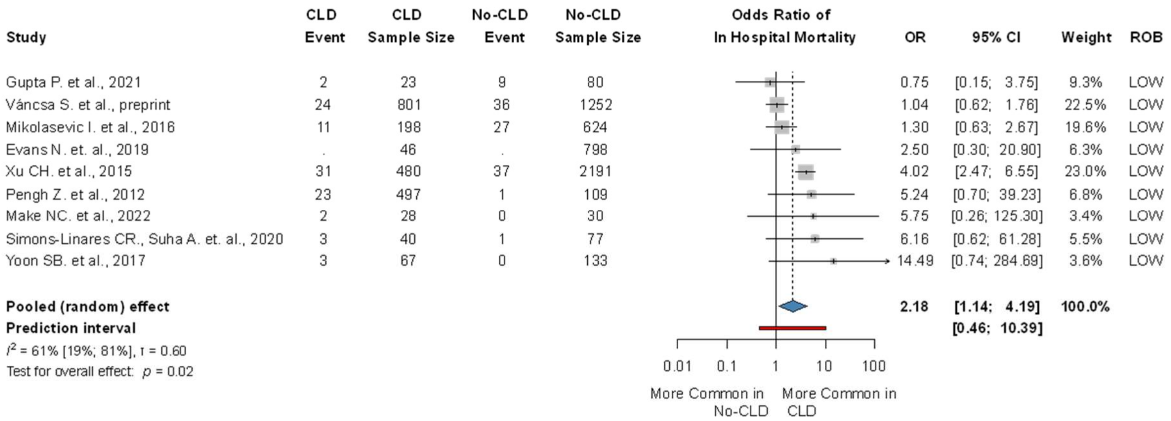

Figure S13: The OR for Organ Failure in patients with AP and with and without CLD in the studies with low ROB.

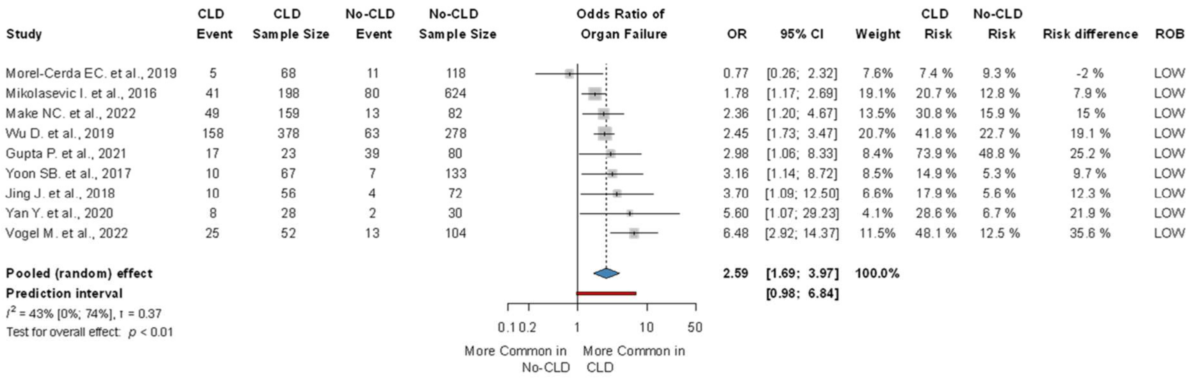

Figure S14: The OR for Systemic complications in patients with AP and with and without CLD in the studies with low ROB.

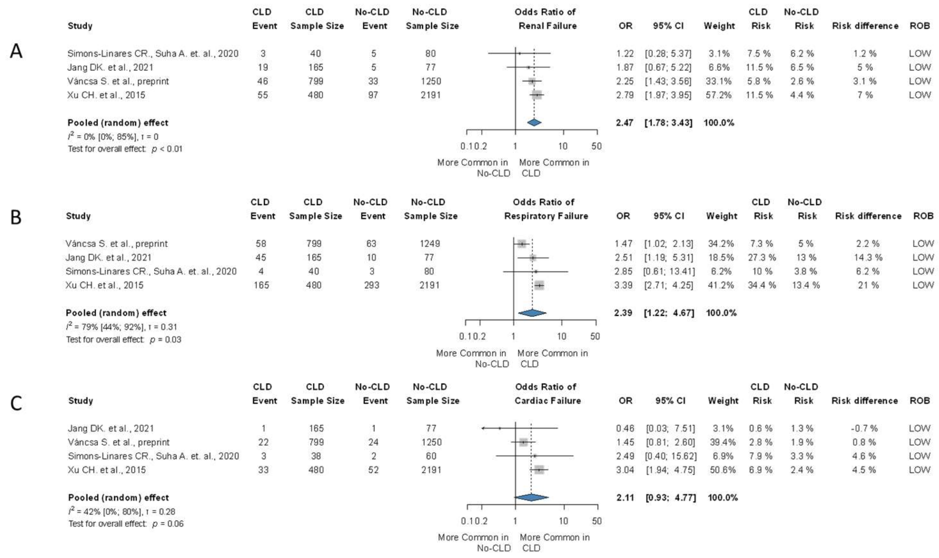

Figure S15: The OR for Local complications in patients with AP and with and without CLD in the studies with low ROB

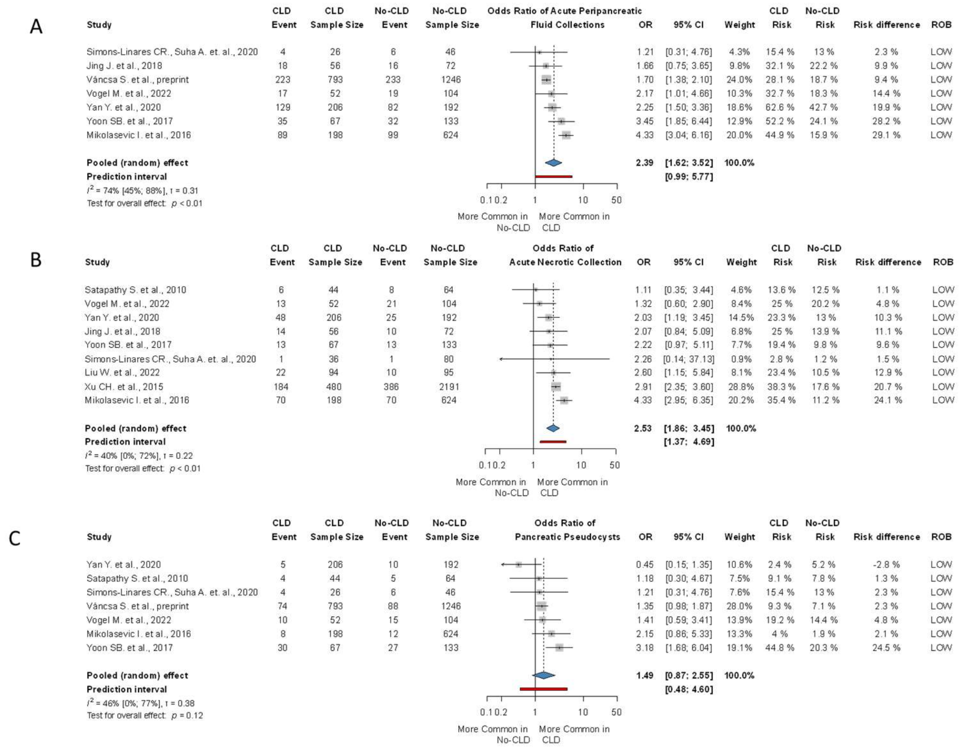

Figure S16: The leave-one-out analysis for the Systemic Inflammatory Response

Study	Influental	effect size	95% CI	I2	Std residual	Dffits	Cook's dist.	Covariance ratio	Hat value
Omitting Liu W. et al., 2022		1.74	[0.86; 3.50]	0.68	1.15	0.45	0.19	1.10	0.15
Omitting Xu CH. et al., 2015		1.77	[0.81; 3.87]	0.68	0.65	0.34	0.14	1.38	0.18
Omitting Yan Y. et al., 2020		1.81	[0.81; 4.04]	0.72	0.41	0.24	0.07	1.44	0.17
Omitting Wu D. et al., 2019		1.81	[0.81; 4.05]	0.72	0.40	0.24	0.07	1.45	0.17
Omitting Wang S. et al., 2013		1.93	[0.87; 4.30]	0.70	-0.14	0.02	0.00	1.44	0.13
Omitting Morel-Cerda EC. et al., 2019		2.19	[1.16; 4.14]	0.62	-1.39	-0.49	0.22	0.91	0.09
Omitting Simons-Linares CR., Suha A. et. al., 2020	*	2.41	[1.52; 3.83]	0.40	-2.35	-1.06	0.69	0.48	0.11
**Pooled (random) effect**		**1.94**	**[1.03; 3.67]**						

I^2 = 66% [25%; 85%]

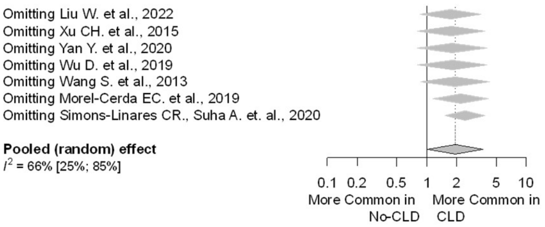

Figure S17: The leave-one-out analysis for Severe Acute Pancreatitis

Study	Influential	effect size	95% CI	I2	Std residual	Dffits	Cook's dist.	Covariance ratio	Hat value
Omitting Xu CH. et al., 2015	*	2.14	[1.70; 2.68]	0.00	1.13	0.79	0.57	1.43	0.32
Omitting Mikolasevic I. et al., 2016		2.24	[1.79; 2.81]	0.06	0.34	0.20	0.05	1.41	0.15
Omitting Liu W. et al., 2022		2.26	[1.84; 2.77]	0.05	0.57	0.14	0.02	1.10	0.04
Omitting Yoon SB. et al., 2017		2.26	[1.85; 2.76]	0.04	0.63	0.13	0.02	1.07	0.03
Omitting Jing J. et al., 2018		2.26	[1.86; 2.75]	0.03	0.78	0.13	0.02	1.05	0.02
Omitting Make NC. et al., 2022		2.26	[1.87; 2.74]	0.00	1.07	0.12	0.01	1.03	0.01
Omitting Park HS. et al., 2019		2.27	[1.84; 2.79]	0.06	0.29	0.10	0.01	1.14	0.05
Omitting Xiao B. et al., 2012		2.27	[1.88; 2.74]	0.00	1.20	0.08	0.01	1.01	0.00
Omitting Wang S. et al., 2013		2.28	[1.85; 2.80]	0.07	0.14	0.06	0.00	1.12	0.05
Omitting Simons-Linares CR., Suha A. et. al., 2020		2.28	[1.86; 2.79]	0.07	0.23	0.05	0.00	1.06	0.02
Omitting Jimenez MJ. et al., 2021		2.30	[1.90; 2.79]	0.02	-0.84	-0.07	0.01	1.00	0.01
Omitting Wu D. et al., 2019		2.31	[1.88; 2.84]	0.05	-0.52	-0.12	0.01	1.12	0.06
Omitting Lupulescu P. et al., 2019		2.31	[1.92; 2.79]	0.00	-1.25	-0.13	0.02	0.99	0.01
Omitting Morel-Cerda EC. et al., 2019		2.37	[2.00; 2.82]	0.00	-2.23	-0.42	0.17	0.99	0.03
Omitting Váncsa S. et al., preprint	*	2.43	[1.99; 2.97]	0.00	-1.44	-0.72	0.49	1.18	0.19
**Pooled (random) effect**		**2.29**	**[1.89; 2.76]**						

I^2 = 0% [0%; 54%]

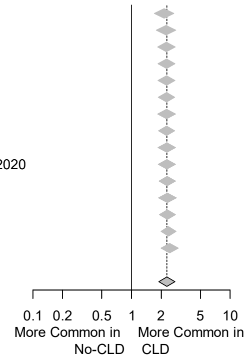

Figure S18: The leave-one-out analysis for In-Hospital Mortality

Study	Influental	effect size	95% CI	I2	Std residual	Dffits	Cook's dist.	Covariance ratio	Hat value
Omitting Brivet F. et al., 1999		2.13	[1.19; 3.83]	0.58	2.07	0.63	0.33	0.78	0.06
Omitting Jasdanwala S. et al., 2015		2.34	[1.18; 4.63]	0.64	1.09	0.27	0.07	1.00	0.05
Omitting Yoon SB. et al., 2017		2.39	[1.21; 4.71]	0.64	1.06	0.20	0.04	1.00	0.03
Omitting Xu CH. et al., 2015		2.39	[1.12; 5.09]	0.53	0.58	0.18	0.04	1.28	0.15
Omitting Simons-Linares CR., Suha A. et. al., 2020		2.43	[1.20; 4.95]	0.65	0.65	0.13	0.02	1.07	0.05
Omitting Pengh Z. et al., 2012		2.44	[1.19; 5.00]	0.65	0.58	0.12	0.02	1.10	0.05
Omitting Make NC. et al., 2022		2.48	[1.22; 5.04]	0.66	0.48	0.07	0.00	1.06	0.03
Omitting Evans N. et. al., 2019		2.57	[1.24; 5.34]	0.66	-0.02	-0.05	0.00	1.15	0.05
Omitting Liu Z et al., 2022		2.65	[1.23; 5.72]	0.66	-0.11	-0.13	0.02	1.39	0.13
Omitting Gupta P. et al., 2021		2.80	[1.40; 5.59]	0.64	-1.14	-0.33	0.11	1.11	0.07
Omitting Zhang X.Ch. et al., 2021		2.81	[1.50; 5.26]	0.62	-1.76	-0.36	0.13	0.98	0.05
Omitting Mikolasevic I. et al., 2016		2.84	[1.35; 5.94]	0.64	-0.84	-0.36	0.15	1.26	0.13
Omitting Váncsa S. et al., preprint		2.91	[1.44; 5.88]	0.51	-1.30	-0.50	0.22	1.07	0.14
**Pooled (random) effect**		**2.53**	**[1.30; 4.93]**						

I^2 = 63% [33%; 80%]

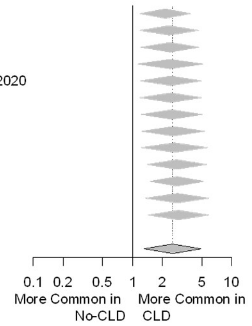

Figure S19: The leave-one-out analysis for Organ Failure in patients

Study	Influential	effect size	95% CI	I2	Std residual	Dffits	Cook's dist.	Covariance ratio	Hat value
Omitting Vogel M. et al., 2022	*	2.24	[1.65; 3.04]	0.08	2.40	1.02	0.62	0.49	0.11
Omitting Yan Y. et al., 2020		2.51	[1.59; 3.98]	0.46	0.85	0.17	0.03	1.10	0.04
Omitting Jing J. et al., 2018		2.54	[1.56; 4.14]	0.48	0.48	0.10	0.01	1.23	0.07
Omitting Yoon SB. et al., 2017		2.56	[1.56; 4.22]	0.49	0.30	0.05	0.00	1.29	0.09
Omitting Gupta P. et al., 2021		2.58	[1.57; 4.26]	0.49	0.20	0.02	0.00	1.30	0.08
Omitting Wu D. et al., 2019		2.65	[1.56; 4.52]	0.50	-0.16	-0.11	0.02	1.47	0.21
Omitting Make NC. et al., 2022		2.66	[1.59; 4.44]	0.50	-0.20	-0.12	0.02	1.37	0.13
Omitting Morel-Cerda EC. et al., 2019		2.69	[1.90; 3.81]	0.28	-2.02	-0.22	0.04	0.63	0.08
Omitting Mikolasevic I. et al., 2016		2.83	[1.74; 4.62]	0.37	-0.99	-0.48	0.23	1.24	0.19
**Pooled (random) effect**		**2.59**	**[1.69; 3.97]**						

I^2 = 43% [0%; 74%]

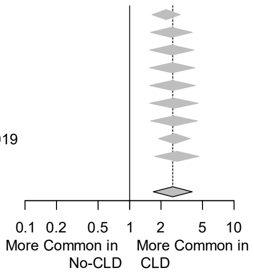

Figure S20: The leave-one-out analysis for Cardiac Failure

Study	Odds Ratio of Cardiac Failure	Influental	effect size	95% CI	I2	Std residual	Dffits	Cook's dist.	Covariance ratio	Hat value
Omitting Xu CH. et al., 2015		*	1.45	[0.63; 3.36]	0.00	2.05	2.29	2.12	1.19	0.51
Omitting Simons-Linares CR., Suha A. et. al., 2020			1.97	[0.42; 9.20]	0.61	0.21	0.26	0.07	1.96	0.07
Omitting Jang DK. et al., 2021			2.22	[0.75; 6.56]	0.49	-1.07	-0.20	0.04	0.97	0.03
Omitting Váncsa S. et al., preprint		*	2.87	[1.19; 6.91]	0.00	-1.85	-1.65	1.45	0.73	0.39
**Pooled (random) effect**			**2.11**	**[0.93; 4.77]**						
I^2 = 42% [0%; 80%]										

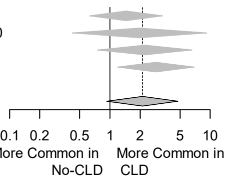

Figure S21: The leave-one-out analysis for Respiratory Failure

Study	Odds Ratio of Respiratory Failure	Influental	effect size	95% CI	I2	Std residual	Dffits	Cook's dist.	Covariance ratio	Hat value
Omitting Xu CH. et al., 2015		*	1.68	[0.83; 3.37]	0.84	1.34	0.78	0.52	1.13	0.22
Omitting Wang S. et al., 2013			1.84	[0.89; 3.79]	0.97	0.84	0.32	0.10	1.22	0.13
Omitting Jang DK. et al., 2021			1.93	[0.89; 4.20]	0.97	0.36	0.11	0.01	1.40	0.15
Omitting Simons-Linares CR., Suha A. et. al., 2020			1.96	[0.93; 4.11]	0.97	0.38	0.06	0.00	1.28	0.06
Omitting Váncsa S. et al., preprint			2.17	[1.00; 4.72]	0.97	-0.62	-0.34	0.13	1.41	0.20
Omitting Shah I. et al., 2020		*	2.50	[1.51; 4.14]	0.73	-3.10	-1.75	0.95	0.59	0.23
**Pooled (random) effect**			**1.99**	**[1.08; 3.65]**						
I^2 = 97% [95%; 98%]										

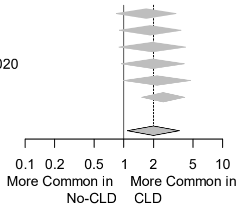

Figure S22: The leave-one-out analysis for Renal Failure

Study	Odds Ratio of Renal Failure	Influental	effect size	95% CI	I2	Std residual	Dffits	Cook's dist.	Covariance ratio	Hat value
Omitting Xu CH. et al., 2015		*	1.53	[1.25; 1.88]	0.14	3.01	2.33	2.59	0.21	0.26
Omitting Váncsa S. et al., preprint			1.86	[1.07; 3.25]	0.77	0.50	0.20	0.05	1.56	0.20
Omitting Jang DK. et al., 2021			1.95	[1.14; 3.33]	0.81	-0.07	-0.12	0.02	1.44	0.06
Omitting Simons-Linares CR., Suha A. et. al., 2020			1.97	[1.20; 3.23]	0.81	-0.60	-0.19	0.04	1.24	0.03
Omitting Simons-Linares CR. et. al., 2019		*	2.47	[1.78; 3.43]	0.00	-3.78	-25.83	3.28	0.92	0.45
**Pooled (random) effect**			**1.92**	**[1.30; 2.83]**						
I^2 = 75% [39%; 90%]										

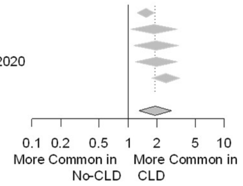

Figure S23: The leave-one-out analysis for Acute Necrotic Collection

Study	Odds Ratio of Acute Necrotic Collection	Influental	effect size	95% CI	I2	Std residual	Dffits	Cook's dist.	Covariance ratio	Hat value
Omitting Xu CH. et al., 2015			2.37	[1.61; 3.49]	0.46	0.65	0.46	0.25	1.50	0.29
Omitting Liu W. et al., 2022			2.47	[1.73; 3.54]	0.47	0.10	0.17	0.03	1.29	0.08
Omitting Simons-Linares CR., Suha A. et. al., 2020			2.49	[1.76; 3.51]	0.48	-0.07	0.14	0.02	1.19	0.01
Omitting Yoon SB. et al., 2017			2.51	[1.76; 3.59]	0.46	-0.23	0.06	0.00	1.27	0.08
Omitting Mikolasevic I. et al., 2016			2.51	[2.01; 3.14]	0.03	2.46	0.08	0.00	0.50	0.20
Omitting Jing J. et al., 2018			2.53	[1.78; 3.59]	0.46	-0.37	0.01	0.00	1.24	0.07
Omitting Yan Y. et al., 2020			2.59	[1.81; 3.71]	0.41	-0.62	-0.17	0.03	1.29	0.14
Omitting Satapathy S. et al., 2010			2.68	[1.99; 3.60]	0.35	-1.42	-0.43	0.17	0.89	0.05
Omitting Vogel M. et al., 2022			2.76	[2.07; 3.68]	0.28	-1.63	-0.67	0.41	0.83	0.08
**Pooled (random) effect**			**2.53**	**[1.86; 3.45]**						
I^2 = 40% [0%; 72%]										

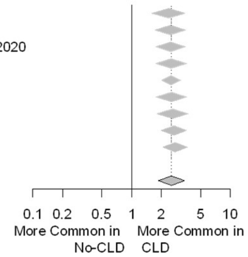

Figure S24: The leave-one-out analysis for Pancreatic Pseudocysts

Study	Odds Ratio of Pancreatic Pseudocysts	Influental	effect size	95% CI	I2	Std residual	Dffits	Cook's dist.	Covariance ratio	Hat value
Omitting Mikolasevic I. et al., 2016			1.47	[0.92; 2.34]	0.45	0.62	0.24	0.06	1.30	0.09
Omitting Shah I. et al., 2020			1.49	[0.87; 2.55]	0.46	0.15	0.13	0.02	1.71	0.29
Omitting Yoon SB. et al., 2017			1.51	[1.24; 1.83]	0.13	2.15	0.13	0.01	0.22	0.14
Omitting Vogel M. et al., 2022			1.53	[0.94; 2.50]	0.47	-0.13	0.01	0.00	1.43	0.09
Omitting Simons-Linares CR., Suha A. et. al., 2020			1.54	[0.96; 2.47]	0.47	-0.29	-0.03	0.00	1.34	0.05
Omitting Satapathy S. et al., 2010			1.54	[0.96; 2.47]	0.46	-0.33	-0.04	0.00	1.33	0.05
Omitting Váncsa S. et al., preprint			1.56	[0.93; 2.63]	0.42	-0.31	-0.11	0.02	1.60	0.23
Omitting Yan Y. et al., 2020			1.60	[1.39; 1.83]	0.04	-2.24	-0.28	0.06	0.11	0.07
**Pooled (random) effect**			**1.53**	**[1.03; 2.27]**						
I^2 = 38% [0%; 73%]										

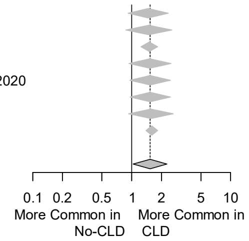

Figure S25: The leave-one-out analysis for Acute Peripancreatic Fluid Collections

Study	Odds Ratio of Acute Peripancreatic Fluid Collections	Influental	effect size	95% CI	I2	Std residual	Dffits	Cook's dist.	Covariance ratio	Hat value
Omitting Mikolasevic I. et al., 2016		*	1.95	[1.49; 2.54]	0.15	3.51	2.24	1.63	0.43	0.20
Omitting Yoon SB. et al., 2017			2.26	[1.45; 3.52]	0.76	0.89	0.35	0.13	1.19	0.13
Omitting Vogel M. et al., 2022			2.40	[1.50; 3.85]	0.78	-0.18	-0.04	0.00	1.34	0.10
Omitting Yan Y. et al., 2020			2.41	[1.47; 3.94]	0.78	-0.15	-0.05	0.00	1.47	0.19
Omitting Simons-Linares CR., Suha A. et. al., 2020			2.46	[1.61; 3.76]	0.78	-0.91	-0.19	0.04	1.08	0.04
Omitting Jing J. et al., 2018			2.48	[1.59; 3.88]	0.78	-0.74	-0.24	0.06	1.21	0.10
Omitting Váncsa S. et al., preprint		*	2.68	[1.72; 4.18]	0.54	-1.32	-0.80	0.54	1.19	0.24
**Pooled (random) effect**			**2.39**	**[1.62; 3.52]**						

I^2 = 74% [45%; 88%]

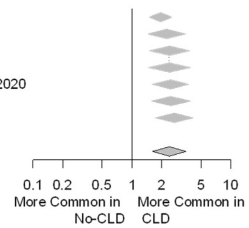

Figure S26: The analysis, including adjusted models for SIRS

Study	CLD Event	CLD Sample Size	No-CLD Event	No-CLD Sample Size	NOT Adjusted OR	Odds Ratio of Systemic Inflammatory Response	OR	95% CI	Weight	ROB	Adjusted for
Simons-Linares CR., Suha A. et. al., 2020	6	40	19	76	0.53		0.53	[0.21; 1.40]	10.2%	LOW	not given
Morel-Cerda EC. et al., 2019	4	68	10	118	0.68		0.68	[0.20; 2.24]	7.7%	LOW	
Wang S. et al., 2013	18	35	32	84	1.72		1.72	[0.78; 3.81]	12.1%	HIGH	
Wu D. et al., 2019	160	378	64	278	2.45		2.45	[1.74; 3.47]	19.2%	LOW	
Yan Y. et al., 2020	120	206	69	192	2.49		2.49	[1.66; 3.73]	18.2%	LOW	
Xu CH. et al., 2015	183	480	392	2191	2.83		2.83	[2.28; 3.50]	20.9%	LOW	
Liu W. et al., 2022	56	94	26	95	3.91		2.86	[1.26; 6.49]	11.8%	LOW	not given
**Pooled (random) effect**							**1.92**	**[1.08; 3.39]**	**100.0%**		
**Prediction interval**								**[0.53; 6.98]**			

I^2 = 65% [21%; 84%], τ = 0.45
Test for overall effect: p = 0.03

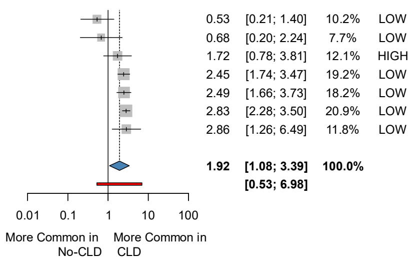

Figure S27: The leave-one-out analysis including adjusted models for SIRS

Study	Odds Ratio of Systemic Inflammatory Response	Influental	effect size	95% CI	I2	Std residual	Dffits	Cook's dist.	Covariance ratio	Hat value
Omitting Xu CH. et al., 2015			1.69	[0.83; 3.43]	0.64	0.88	0.52	0.35	1.52	0.21
Omitting Wu D. et al., 2019			1.73	[0.83; 3.62]	0.71	0.54	0.39	0.23	1.70	0.19
Omitting Yan Y. et al., 2020			1.74	[0.83; 3.61]	0.71	0.55	0.39	0.22	1.68	0.18
Omitting Liu W. et al., 2022			1.75	[0.87; 3.52]	0.70	0.68	0.39	0.18	1.45	0.12
Omitting Wang S. et al., 2013			1.88	[0.91; 3.89]	0.69	-0.12	0.09	0.01	1.51	0.12
Omitting Morel-Cerda EC. et al., 2019			2.20	[1.31; 3.71]	0.60	-1.69	-0.74	0.44	0.61	0.08
Omitting Simons-Linares CR., Suha A. et. al., 2020		*	2.58	[2.03; 3.27]	0.25	-3.22	-1.92	1.99	0.15	0.10
**Pooled (random) effect**			**1.92**	**[1.08; 3.39]**						

I^2 = 65% [21%; 84%]

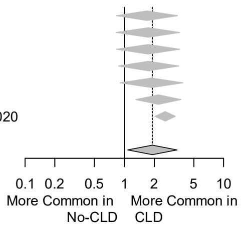

Figure S28: The analysis, including adjusted models for Organ Failure

Study	CLD Event	CLD Sample Size	No-CLD Event	No-CLD Sample Size	NOT Adjusted OR	Odds Ratio of Organ Failure	OR	95% CI	Weight	ROB	Adjusted for
Morel-Cerda EC. et al., 2019	5	68	11	118	0.77		0.77	[0.26; 2.32]	8.0%	LOW	
Mikolasevic I. et al., 2016	41	198	80	624	1.78		1.78	[1.17; 2.69]	20.3%	LOW	
Make NC. et al., 2022	49	159	13	82	2.36		2.36	[1.20; 4.67]	14.2%	LOW	
Gupta P. et al., 2021	17	23	39	80	2.98		2.98	[1.06; 8.33]	8.8%	LOW	
Wu D. et al., 2019	158	378	63	278	2.45		3.12	[1.77; 5.49]	16.6%	LOW	not given
Yoon SB. et al., 2017	10	67	7	133	3.16		3.16	[1.14; 8.72]	8.9%	LOW	
Jing J. et al., 2018	10	56	4	72	3.70		3.70	[1.09; 12.50]	6.9%	LOW	
Yan Y. et al., 2020	8	28	2	30	5.60		5.60	[1.07; 29.23]	4.2%	LOW	
Vogel M. et al., 2022	25	52	13	104	6.48		6.48	[2.92; 14.37]	12.1%	LOW	
**Pooled (random) effect**							**2.70**	**[1.74; 4.20]**	**100.0%**		
**Prediction interval**								**[1.03; 7.12]**			

I^2 = 46% [0%; 75%], τ = 0.36
Test for overall effect: p < 0.01

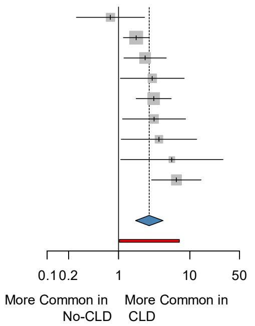

Figure S29: The leave-one-out analysis analysis, including adjusted models for Organ Failure

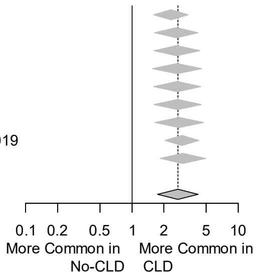

Study	Influental	effect size	95% CI	I2	Std residual	Dffits	Cook's dist.	Covariance ratio	Hat value
Omitting Vogel M. et al., 2022	*	2.33	[1.60; 3.38]	0.19	2.19	0.97	0.62	0.67	0.12
Omitting Yan Y. et al., 2020		2.62	[1.63; 4.20]	0.49	0.81	0.17	0.03	1.06	0.04
Omitting Jing J. et al., 2018		2.65	[1.61; 4.36]	0.51	0.44	0.11	0.01	1.16	0.07
Omitting Wu D. et al., 2019		2.66	[1.56; 4.52]	0.50	0.28	0.08	0.01	1.43	0.17
Omitting Yoon SB. et al., 2017		2.68	[1.61; 4.46]	0.52	0.24	0.05	0.00	1.24	0.09
Omitting Gupta P. et al., 2021		2.69	[1.61; 4.49]	0.52	0.14	0.02	0.00	1.24	0.09
Omitting Make NC. et al., 2022		2.79	[1.65; 4.72]	0.52	-0.28	-0.15	0.03	1.40	0.14
Omitting Morel-Cerda EC. et al., 2019		2.96	[2.05; 4.28]	0.30	-2.03	-0.50	0.23	0.91	0.08
Omitting Mikolasevic I. et al., 2016		3.01	[1.84; 4.92]	0.34	-1.23	-0.63	0.33	1.12	0.20
**Pooled (random) effect**		**2.70**	**[1.74; 4.20]**						

I^2 = 46% [0%; 75%]

Figure S30: The analysis, including adjusted model_1 for Severe Acute Pancreatitis

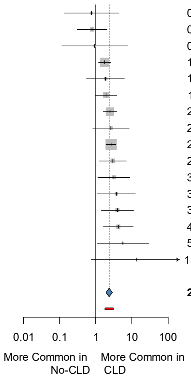

Study	CLD Event	CLD Sample Size	No-CLD Event	No-CLD Sample Size	NOT Adjusted OR	OR	95% CI	Weight	ROB	Adjusted for
Lupulescu P. et al., 2019	2	59	4	91	0.76	0.76	[0.14; 4.30]	1.1%	HIGH	
Morel-Cerda EC. et al., 2019	7	68	15	118	0.79	0.79	[0.30; 2.04]	3.6%	LOW	
Jimenez MJ. et al., 2021	1	11	21	216	0.93	0.93	[0.11; 7.62]	0.8%	HIGH	
Váncsa S. et al., preprint	56	801	51	1252	1.77	1.77	[1.20; 2.62]	19.1%	LOW	
Park HS. et al., 2019	10	63	42	609	2.55	1.86	[0.55; 6.24]	2.3%	HIGH	not given
Wu D. et al., 2019	30	378	12	278	1.91	1.91	[0.96; 3.80]	6.8%	LOW	
Mikolasevic I. et al., 2016	41	198	60	624	2.45	2.45	[1.59; 3.79]	15.8%	LOW	
Simons-Linares CR., Suha A. et. al., 2020	7	40	6	80	2.62	2.62	[0.82; 8.39]	2.4%	LOW	
Xu CH. et al., 2015	.	480	.	2191	2.63	2.63	[1.96; 3.55]	30.0%	LOW	not adjusted
Liu W. et al., 2022	20	94	8	95	2.94	2.94	[1.22; 7.06]	4.3%	LOW	
Yoon SB. et al., 2017	10	67	7	133	3.16	3.16	[1.14; 8.72]	3.2%	LOW	
Jing J. et al., 2018	10	56	4	72	3.70	3.70	[1.09; 12.50]	2.2%	LOW	
Wang S. et al., 2013	22	35	35	85	2.42	3.95	[1.43; 10.93]	3.2%	HIGH	age, sex, etiology
Jang DK. et al., 2021	.	.	.	.		4.19	[1.62; 10.85]	3.6%	LOW	age, sex, etiology, CRP, CTSI
Make NC. et al., 2022	8	28	2	30	5.60	5.60	[1.07; 29.23]	1.2%	LOW	
Xiao B. et al., 2012	9	33	0	17	13.57	13.57	[0.74; 248.93]	0.4%	LOW	
**Pooled (random) effect**						**2.34**	**[1.90; 2.87]**	**100.0%**		
**Prediction interval**							**[1.79; 3.06]**			

I^2 = 10% [0%; 47%], τ = 0.08

Test for overall effect: $p < 0.01$

Figure S31: The leave-one-out analysis, including adjusted model_1 for Severe Acute Pancreatitis

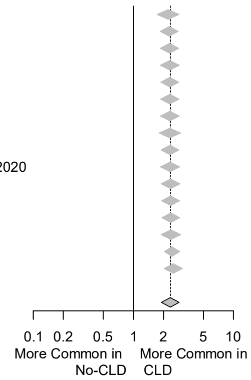

Study	Influental	effect size	95% CI	I2	Std residual	Dffits	Cook's dist.	Covariance ratio	Hat value
Omitting Xu CH. et al., 2015	*	2.23	[1.73; 2.87]	0.11	0.80	0.51	0.27	1.47	0.30
Omitting Jang DK. et al., 2021		2.29	[1.86; 2.82]	0.07	1.21	0.24	0.06	1.04	0.04
Omitting Wang S. et al., 2013		2.30	[1.86; 2.84]	0.10	1.01	0.19	0.04	1.05	0.03
Omitting Liu W. et al., 2022		2.31	[1.85; 2.89]	0.14	0.51	0.12	0.01	1.11	0.04
Omitting Make NC. et al., 2022		2.31	[1.88; 2.85]	0.10	1.04	0.12	0.01	1.02	0.01
Omitting Jing J. et al., 2018		2.31	[1.87; 2.87]	0.13	0.74	0.12	0.01	1.05	0.02
Omitting Yoon SB. et al., 2017		2.31	[1.86; 2.88]	0.14	0.58	0.11	0.01	1.08	0.03
Omitting Mikolasevic I. et al., 2016		2.32	[1.80; 2.98]	0.15	0.20	0.09	0.01	1.49	0.16
Omitting Xiao B. et al., 2012		2.32	[1.89; 2.85]	0.08	1.19	0.08	0.01	1.01	0.00
Omitting Simons-Linares CR., Suha A. et. al., 2020		2.33	[1.87; 2.90]	0.15	0.19	0.03	0.00	1.07	0.02
Omitting Park HS. et al., 2019		2.35	[1.89; 2.92]	0.15	-0.37	-0.05	0.00	1.06	0.02
Omitting Jimenez MJ. et al., 2021		2.36	[1.91; 2.91]	0.12	-0.86	-0.08	0.01	1.01	0.01
Omitting Lupulescu P. et al., 2019		2.37	[1.93; 2.90]	0.06	-1.27	-0.13	0.02	1.00	0.01
Omitting Wu D. et al., 2019		2.37	[1.89; 2.98]	0.14	-0.57	-0.16	0.03	1.16	0.07
Omitting Morel-Cerda EC. et al., 2019		2.43	[2.04; 2.90]	0.00	-2.28	-0.42	0.17	0.90	0.04
Omitting Váncsa S. et al., preprint	*	2.51	[2.03; 3.09]	0.01	-1.57	-0.80	0.55	1.09	0.19
**Pooled (random) effect**		**2.34**	**[1.90; 2.87]**						

I^2 = 10% [0%; 47%]

Figure S32: The analysis, including adjusted models for Severe Acute Pancreatitis

Study	CLD Event	CLD Sample Size	No-CLD Event	No-CLD Sample Size	NOT Adjusted OR	OR	95% CI	Weight	ROB	Adjusted for
Lupulescu P. et al., 2019	2	59	4	91	0.76	0.76	[0.14; 4.30]	1.3%	HIGH	
Morel-Cerda EC. et al., 2019	7	68	15	118	0.79	0.79	[0.30; 2.04]	4.0%	LOW	
Jimenez MJ. et al., 2021	1	11	21	216	0.93	0.93	[0.11; 7.62]	0.9%	HIGH	
Jang DK. et al., 2021	.	.	.	.		1.30	[0.69; 2.44]	8.4%	LOW	not well defined
Váncsa S. et al., preprint	56	801	51	1252	1.77	1.77	[1.20; 2.62]	17.4%	LOW	
Park HS. et al., 2019	10	63	42	609	2.55	1.86	[0.55; 6.24]	2.6%	HIGH	not given
Wu D. et al., 2019	30	378	12	278	1.91	1.91	[0.96; 3.80]	7.2%	LOW	
Mikolasevic I. et al., 2016	41	198	60	624	2.45	2.45	[1.59; 3.79]	15.0%	LOW	
Simons-Linares CR., Suha A. et. al., 2020	7	40	6	80	2.62	2.62	[0.82; 8.39]	2.8%	LOW	
Xu CH. et al., 2015	.	480	.	2191	2.63	2.63	[1.96; 3.55]	24.1%	LOW	not adjusted
Liu W. et al., 2022	20	94	8	95	2.94	2.94	[1.22; 7.06]	4.7%	LOW	
Yoon SB. et al., 2017	10	67	7	133	3.16	3.16	[1.14; 8.72]	3.6%	LOW	
Jing J. et al., 2018	10	56	4	72	3.70	3.70	[1.09; 12.50]	2.5%	LOW	
Wang S. et al., 2013	22	35	35	85	2.42	3.95	[1.43; 10.93]	3.6%	HIGH	age, sex, etiology
Make NC. et al., 2022	8	28	2	30	5.60	5.60	[1.07; 29.23]	1.4%	LOW	
Xiao B. et al., 2012	9	33	0	17	13.57	13.57	[0.74; 248.93]	0.5%	LOW	
**Pooled (random) effect**						**2.17**	**[1.74; 2.71]**	**100.0%**		
**Prediction interval**							**[1.49; 3.17]**			

I^2 = 17% [0%; 54%], τ = 0.14
Test for overall effect: p < 0.01

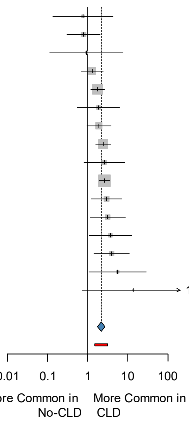

Figure S33: The leave-one-out analysis, including adjusted models for Severe Acute Pancreatitis Table S1: Traffic light plot of the risk of bias

Study	Influental	effect size	95% CI	I2	Std residual	Dffits	Cook's dist.	Covariance ratio	Hat value
Omitting Xu CH. et al., 2015	*	2.03	[1.59; 2.60]	0.13	1.29	0.80	0.43	1.13	0.24
Omitting Mikolasevic I. et al., 2016		2.12	[1.63; 2.75]	0.21	0.47	0.23	0.06	1.38	0.15
Omitting Wang S. et al., 2013		2.12	[1.69; 2.67]	0.16	1.13	0.23	0.05	1.06	0.04
Omitting Liu W. et al., 2022		2.14	[1.68; 2.71]	0.21	0.66	0.17	0.03	1.11	0.05
Omitting Yoon SB. et al., 2017		2.14	[1.69; 2.71]	0.20	0.71	0.15	0.02	1.09	0.04
Omitting Jing J. et al., 2018		2.14	[1.70; 2.70]	0.19	0.84	0.15	0.02	1.06	0.03
Omitting Make NC. et al., 2022		2.14	[1.71; 2.68]	0.17	1.12	0.14	0.02	1.03	0.01
Omitting Xiao B. et al., 2012		2.15	[1.73; 2.69]	0.15	1.23	0.09	0.01	1.01	0.00
Omitting Simons-Linares CR., Suha A. et. al., 2020		2.16	[1.70; 2.74]	0.22	0.31	0.07	0.00	1.08	0.03
Omitting Park HS. et al., 2019		2.18	[1.72; 2.76]	0.22	-0.25	-0.03	0.00	1.06	0.03
Omitting Jimenez MJ. et al., 2021		2.19	[1.74; 2.75]	0.19	-0.79	-0.07	0.01	1.01	0.01
Omitting Wu D. et al., 2019		2.19	[1.71; 2.80]	0.22	-0.34	-0.07	0.01	1.18	0.07
Omitting Lupulescu P. et al., 2019		2.20	[1.77; 2.75]	0.16	-1.18	-0.14	0.02	1.00	0.01
Omitting Morel-Cerda EC. et al., 2019		2.27	[1.87; 2.75]	0.00	-2.11	-0.45	0.19	0.87	0.04
Omitting Váncsa S. et al., preprint		2.28	[1.79; 2.90]	0.15	-0.98	-0.48	0.21	1.14	0.17
Omitting Jang DK. et al., 2021		2.29	[1.86; 2.82]	0.07	-1.65	-0.54	0.26	0.88	0.08
**Pooled (random) effect**		**2.17**	**[1.74; 2.71]**						

I^2 = 17% [0%; 54%]

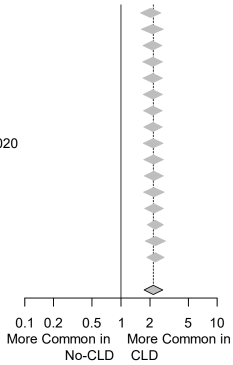

Table S1: Traffic light plot of the risk of bias

Study	D1	D2	D3	D4	D5	D6	Overall
	Risk of bias domains						
Xu Ch. et al.,2015	+	+	+	+	+	+	+
Liu Z et al.,2022	+	+	X	+	+	+	X
Liu W. et al.,2022	+	+	+	+	?	+	+
Brivet F. et al.,1999	+	+	X	+	?	X	X
Ding L. et al.,2018	-	+	X	+	?	+	X
Frey C.et al.,2007	+	?	+	-	?	X	-
Gupta P.et al.,2021	+	+	+	+	+	+	+
Jang DK. et al.,2021	+	+	+	+	+	+	+
Mikolasevic I. et al.,2016	+	+	+	+	+	+	+
Park HS. et al.,2019	+	+	X	+	+	+	-
Roussey B. et al.2022	+	+	X	X	-	+	X
Wu D. et al.,2019	+	+	+	+	+	+	+
Yoon SB. et al.,2017	+	+	+	+	+	+	+
Zhang X.Ch.et al.,2021	+	?	X	+	+	+	X
Xiao B. et al.,2012	+	?	+	+	+	+	+
Jasdanwala S.et al.,2015	+	?	+	+	+	-	-
Simons-Linares CR.,Carlos RM.,et. al.2020	-	?	-	+	+	+	-
Simons-Linares CR.,Suha A.,et. al.2020	+	+	+	+	+	+	+
Vogel M. et al.,2022	+	+	+	+	+	+	+
Husu HL. et al.,2019	+	?	X	+	-	+	X
Jing J. et al.,2018	+	?	+	+	+	-	+
Morel-Cerda EC. et al.,2019	+	?	+	+	+	+	+
Dou J. et al.,2017	+	?	-	+	?	+	+
Yan Y. et al.,2020	+	?	+	+	?	+	+
Váncsa S. et al., pre print	+	+	+	+	+	+	+
Pengh Z. et al.,2012	+	?	+	+	?	+	+
Simons-Linares CR. et. al.,2019	+	?	-	+	?	?	-
Jimenez MJ. et al., 2021	+	?	-	+	?	+	-
Lupulescu P. et al.,2019	-	?	X	-	?	?	X
Mahfouz R. et. at.,2022	-	?	-	+	?	+	-
Evans N. et al.,2019	+	?	-	+	?	?	+
Simons-Linares CR.,Chittajallu V., et. al.,2019	+	?	X	-	?	?	X
Wang S. et al.,2013	+	?	-	-	?	?	-
Shah I. et al.,2020	+	?	-	-	+	?	-
Make NC. et al.,2022	?	?	+	+	?	?	+
Satapathy S. et al.,2010	+	?	+	+	?	?	+

Domains:
D1: Bias due to participation
D2: Bias due to attrition
D3: Bias due to prognostic factor measurement
D4: Bias due to outcome measurement
D5: Bias due to confounding
D6: Bias in statistical analysis and reporting

Judgement: Red: high; Yellow: Moderate; Green: Low; Blue: No information

Table S2: PRISMA checklist[12]

Section and Topic	Item #	Checklist item	The location where item is reported
**TITLE**			
Title	1	Identify the report as a systematic review.	1
**ABSTRACT**			
Abstract	2	See the PRISMA 2020 for Abstracts checklist.	3
**INTRODUCTION**			
Rationale	3	Describe the rationale for the review in the context of existing knowledge.	5
Objectives	4	Provide an explicit statement of the objective(s) or question(s) the review addresses.	5
**METHODS**			
Eligibility criteria	5	Specify the inclusion and exclusion criteria for the review and how studies were grouped for the syntheses.	6
Information sources	6	Specify all databases, registers, websites, organisations, reference lists and other sources searched or consulted to identify studies. Specify the date when each source was last searched or consulted.	6
Search strategy	7	Present the full search strategies for all databases, registers and websites, including any filters and limits used.	6
Selection process	8	Specify the methods used to decide whether a study met the inclusion criteria of the review, including how many reviewers screened each record and each report retrieved, whether they worked independently, and if applicable, details of automation tools used in the process.	6
Data collection process	9	Specify the methods used to collect data from reports, including how many reviewers collected data from each report, whether they worked independently, any processes for obtaining or confirming data from study investigators, and if applicable, details of automation tools used in the process.	6
Data items	10a	List and define all outcomes for which data were sought. Specify whether all results that were compatible with each outcome domain in each study were sought (e.g. for all measures, time points, analyses), and if not, the methods used to decide which results to collect.	6
	10b	List and define all other variables for which data were sought (e.g. participant and intervention characteristics, funding sources). Describe any assumptions made about any missing or unclear information.	6
Study risk of bias assessment	11	Specify the methods used to assess risk of bias in the included studies, including details of the tool(s) used, how many reviewers assessed each study and whether they worked independently, and if applicable, details of automation tools used in the process.	6
Effect measures	12	Specify for each outcome the effect measure(s) (e.g. risk ratio, mean difference) used in the synthesis or presentation of results.	6
Synthesis methods	13a	Describe the processes used to decide which studies were eligible for each synthesis (e.g. tabulating the study intervention characteristics and comparing against the planned groups for each synthesis (item #5)).	7
	13b	Describe any methods required to prepare the data for presentation or synthesis, such as handling of missing summary statistics, or data conversions.	7
	13c	Describe any methods used to tabulate or visually display results of individual studies and syntheses.	7
	13d	Describe any methods used to synthesize results and provide a rationale for the choice(s). If meta-analysis was performed, describe the	7+

Section and Topic	Item #	Checklist item	The location where item is reported
		model(s), method(s) to identify the presence and extent of statistical heterogeneity, and software package(s) used.	Supplementary
	13e	Describe any methods used to explore possible causes of heterogeneity among study results (e.g. subgroup analysis, meta-regression).	Supplementary
	13f	Describe any sensitivity analyses conducted to assess robustness of the synthesized results.	Supplementary
Reporting bias assessment	14	Describe any methods used to assess risk of bias due to missing results in a synthesis (arising from reporting biases).	7
Certainty assessment	15	Describe any methods used to assess certainty (or confidence) in the body of evidence for an outcome.	7
**RESULTS**			
Study selection	16a	Describe the results of the search and selection process, from the number of records identified in the search to the number of studies included in the review, ideally using a flow diagram.	8
	16b	Cite studies that might appear to meet the inclusion criteria, but which were excluded, and explain why they were excluded.	8
Study characteristics	17	Cite each included study and present its characteristics.	8
Risk of bias in studies	18	Present assessments of risk of bias for each included study.	Supplementary
Results of individual studies	19	For all outcomes, present, for each study: (a) summary statistics for each group (where appropriate) and (b) an effect estimate and its precision (e.g. confidence/credible interval), ideally using structured tables or plots.	7-8
Results of syntheses	20a	For each synthesis, briefly summarise the characteristics and risk of bias among contributing studies.	Supplementary
	20b	Present results of all statistical syntheses conducted. If meta-analysis was done, present for each the summary estimate and its precision (e.g. confidence/credible interval) and measures of statistical heterogeneity. If comparing groups, describe the direction of the effect.	8-9
	20c	Present results of all investigations of possible causes of heterogeneity among study results.	Supplementary
	20d	Present results of all sensitivity analyses conducted to assess the robustness of the synthesized results.	Supplementary
Reporting biases	21	Present assessments of risk of bias due to missing results (arising from reporting biases) for each synthesis assessed.	Supplementary
Certainty of evidence	22	Present assessments of certainty (or confidence) in the body of evidence for each outcome assessed.	Supplementary
**DISCUSSION**			
Discussion	23a	Provide a general interpretation of the results in the context of other evidence.	10-11
	23b	Discuss any limitations of the evidence included in the review.	12
	23c	Discuss any limitations of the review processes used.	12
	23d	Discuss implications of the results for practice, policy, and future research.	12
**OTHER INFORMATION**			

Section and Topic	Item #	Checklist item	The location where item is reported
Registration and protocol	24a	Provide registration information for the review, including register name and registration number, or state that the review was not registered.	6
	24b	Indicate where the review protocol can be accessed, or state that a protocol was not prepared.	6
	24c	Describe and explain any amendments to information provided at registration or in the protocol.	Supplementary
Support	25	Describe sources of financial or non-financial support for the review, and the role of the funders or sponsors in the review.	2
Competing interests	26	Declare any competing interests of review authors.	2
Availability of data, code and other materials	27	Report which of the following are publicly available and where they can be found: template data collection forms; data extracted from included studies; data used for all analyses; analytic code; any other materials used in the review.	2

From: Page MJ, McKenzie JE, Bossuyt PM, Boutron I, Hoffmann TC, Mulrow CD, et al. The PRISMA 2020 statement: an updated guideline for reporting systematic reviews. BMJ 2021;372:n71. doi: 10.1136/bmj.n71

For more information, visit: http://www.prisma-statement.org/

Table S3: GRADEpro assessment of the level of evidence

Certainty assessment							№ of patients		Effect		Certainty	Importance
№ of studies	Study design	Risk of bias	Inconsistency	Indirectness	Imprecision	Other considerations	presence of CLD	absence	Relative (95% CI)	Absolute (95% CI)		
**Mortality (assessed with: OR)**												
12	observational studies	not serious	serious	not serious	not serious	strong association	-/2451	-/6512	**OR 2.57** (1.31 to 5.04)	**0 fewer per 100** (from 0 fewer to 0 fewer)	⨁⨁◯◯ Low	
**Severe acute pancreatitis (assessed with: OR)**												
10	observational studies	not serious	not serious	not serious	not serious	strong association	-/1063	-/5891	**OR 2.29** (1.90 to 2.76)	**0 fewer per 100** (from 0 fewer to 0 fewer)	⨁⨁⨁◯ Moderate	
**Organ failure (assessed with: OR)**												
9	observational studies	serious [a]	not serious	not serious	not serious	strong association	323/1029 (31.4%)	232/1521 (15.3%)	**OR 2.59** (1.69 to 3.97)	**17 more per 100** (from 8 more to 26 more)	⨁⨁◯◯ Low	
**SIRS (assessed with: OR)**												
7	observational studies	not serious	serious [b]	not serious	serious [c]	strong association	547/1301 (42.0%)	612/3035 (20.2%)	**OR 1.95** (1.03 to 3.68)	**13 more per 100** (from 0 fewer to 28 more)	⨁◯◯◯ Very low	
**ANC (assessed with: OR)**												
9	observational studies	not serious	not serious	not serious	not serious	strong association	371/1233 (30.1%)	544/3555 (15.3%)	**OR 2.53** (1.86 to 3.45)	**161 more per 1 000** (from 98 more to 231 more)	⨁⨁⨁◯ Moderate	

OR: odds ratio; CI: confidence interval; CLD: chronic liver diseases

References

1 Mantel, N. & Haenszel, W. Statistical Aspects of the Analysis of Data From Retrospective Studies of Disease. *JNCI: Journal of the National Cancer Institute* **22**, 719-748, doi:10.1093/jnci/22.4.719 (1959).

2 Robins, J., Greenland, S. & Breslow, N. E. A general estimator for the variance of the Mantel-Haenszel odds ratio. *Am J Epidemiol* **124**, 719-723, doi:10.1093/oxfordjournals.aje.a114447 (1986).

3 Cooper, H., Hedges, L. V. & Valentine, J. C. *The Handbook of Research Synthesis and Meta-Analysis, Second Edition*. (Russell Sage Foundation, 2009).

4 Sweeting, M. J., Sutton, A. J. & Lambert, P. C. What to add to nothing? Use and avoidance of continuity corrections in meta-analysis of sparse data. *Stat Med* **23**, 1351-1375, doi:10.1002/sim.1761 (2004).

5 Knapp, G. & Hartung, J. Improved tests for a random effects meta-regression with a single covariate. *Stat Med* **22**, 2693-2710, doi:10.1002/sim.1482 (2003).

6 IntHout, J., Ioannidis, J. P. A. & Borm, G. F. The Hartung-Knapp-Sidik-Jonkman method for random effects meta-analysis is straightforward and considerably outperforms the standard DerSimonian-Laird method. *BMC Medical Research Methodology* **14**, 25, doi:10.1186/1471-2288-14-25 (2014).

7 Jackson, D., Law, M., Rücker, G. & Schwarzer, G. The Hartung-Knapp modification for random-effects meta-analysis: A useful refinement but are there any residual concerns? *Statistics in Medicine* **36**, 3923-3934, doi:https://doi.org/10.1002/sim.7411 (2017).

8 Paule, R. C. & Mandel, J. Consensus Values and Weighting Factors. *J Res Natl Bur Stand (1977)* **87**, 377-385, doi:10.6028/jres.087.022 (1982).

9 Veroniki, A. A. *et al.* Methods to estimate the between-study variance and its uncertainty in meta-analysis. *Res Synth Methods* **7**, 55-79, doi:10.1002/jrsm.1164 (2016).

10 Mathias Harrer, P. C., Toshi Furukawa, David Ebert. Doing Meta-Analysis with RA Hands-On Guide. doi:https://doi.org/10.1201/9781003107347 (2021).

11 Harbord, R. M., Egger, M. & Sterne, J. A. A modified test for small-study effects in meta-analyses of controlled trials with binary endpoints. *Stat Med* **25**, 3443-3457, doi:10.1002/sim.2380 (2006).

12 Page, M. J. *et al.* The PRISMA 2020 statement: an updated guideline for reporting systematic reviews. *BMJ* **372**, n71, doi:10.1136/bmj.n71 (2021).
